# Supplementary material for: Differential damage and repair of DNA-adducts induced by anti-cancer drug cisplatin across mouse organs
Source: Nat Commun. 2019 Jan 18;10:309. doi: 10.1038/s41467-019-08290-2 (PMC6338751; doi:10.1038/s41467-019-08290-2)
Supplement: Supplementary file 1 — Supplementary Information [file 41467_2019_8290_MOESM1_ESM.pdf]

Differential damage and repair of DNA adducts introduced by anti-cancer drug cisplatin across mouse organs

Yimit *et al.*

Table of Contents

Supplementary Figure 1. 3
Supplementary Figure 2. 4
Supplementary Figure 3. 5
Supplementary Figure 4. 6
Supplementary Figure 5. 7
Supplementary Figure 6. 8
Supplementary Figure 7. 9
Supplementary Figure 8. 10
Supplementary Figure 9. 11
Supplementary Figure 10. 12
Supplementary Figure 11. 13
Supplementary Figure 12. 14
Supplementary Figure 13. 15
Supplementary Figure 14. 17
Supplementary Figure 15. 18
Supplementary Figure 16. 22
Supplementary Figure 17. 26
Supplementary Figure 18. 27
Supplementary Figure 19. 28
Supplementary Table 1. 29

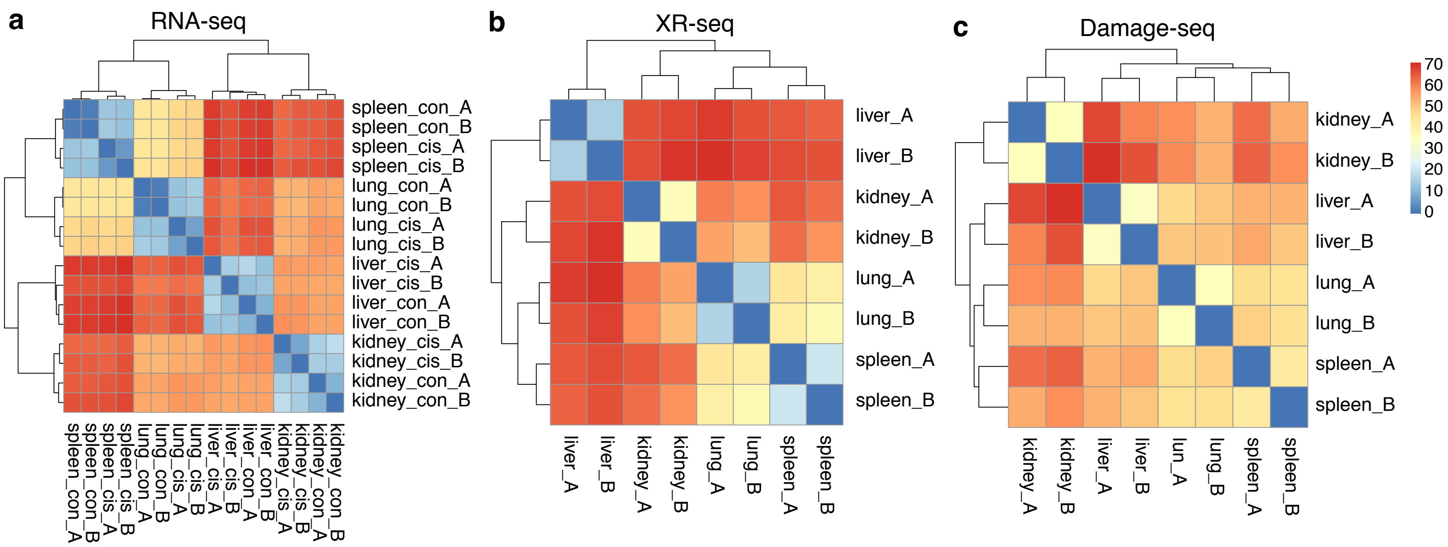

Supplementary Figure 1. Hierarchical clustering of gene expression, excision repair, and damage. Pairwise Euclidean distance is calculated between each sample-pair using the normalized read counts, followed by hierarchical clustering. Samples from the same organ clustered together. **a** Indicating tissue-specific transcription. **b** Indicating tissue-specific excision repair. **c** Indicating tissue-specific damage. For RNA-seq, controls and cases form sub-clusters within each organ.

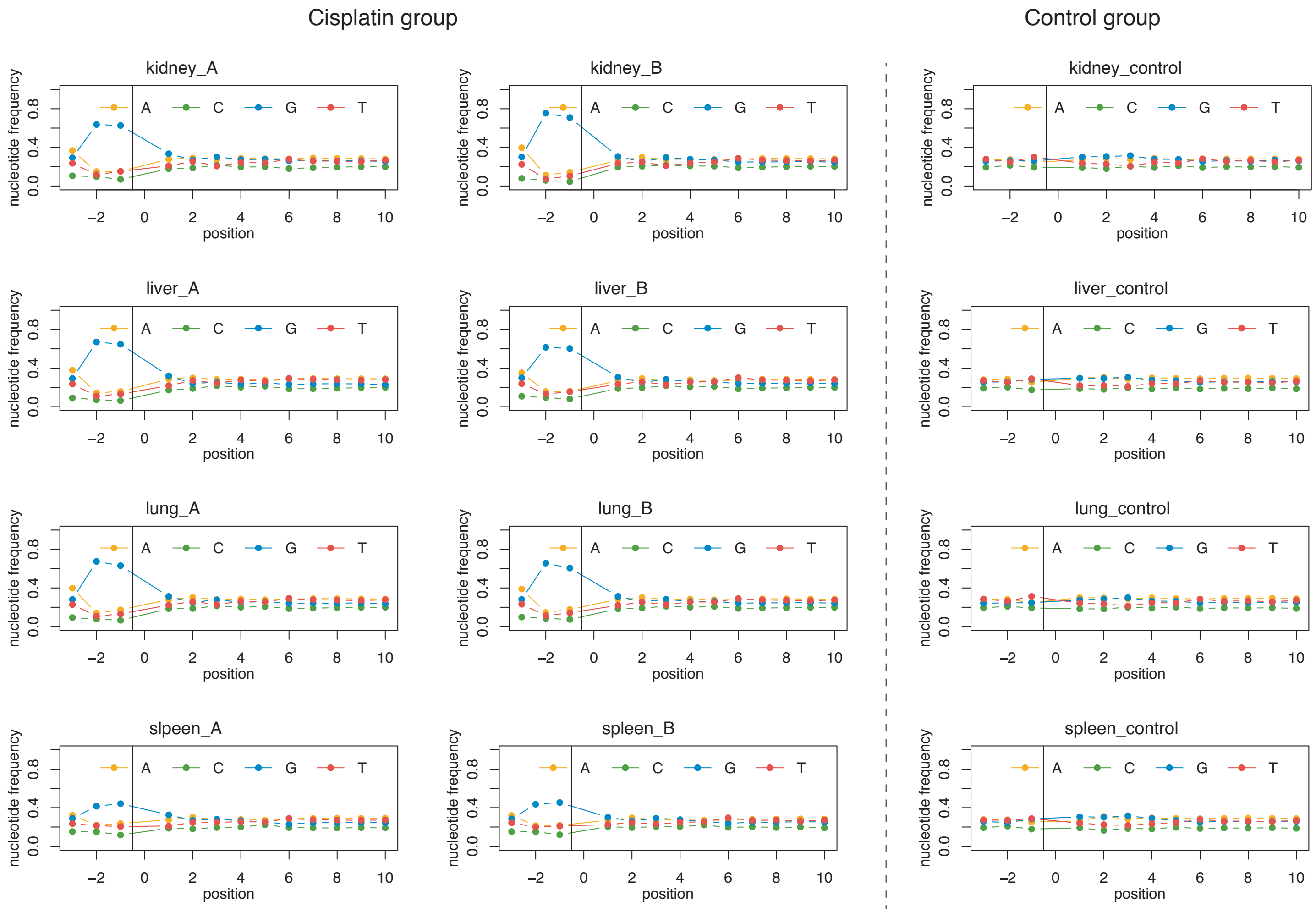

Supplementary Figure 2. Nucleotide frequency from Damage-seq. GG dinucleotide is enriched 1-2 bp upstream of the 5' end of the reads from the cisplatin group. No enrichment is observed in the control group.

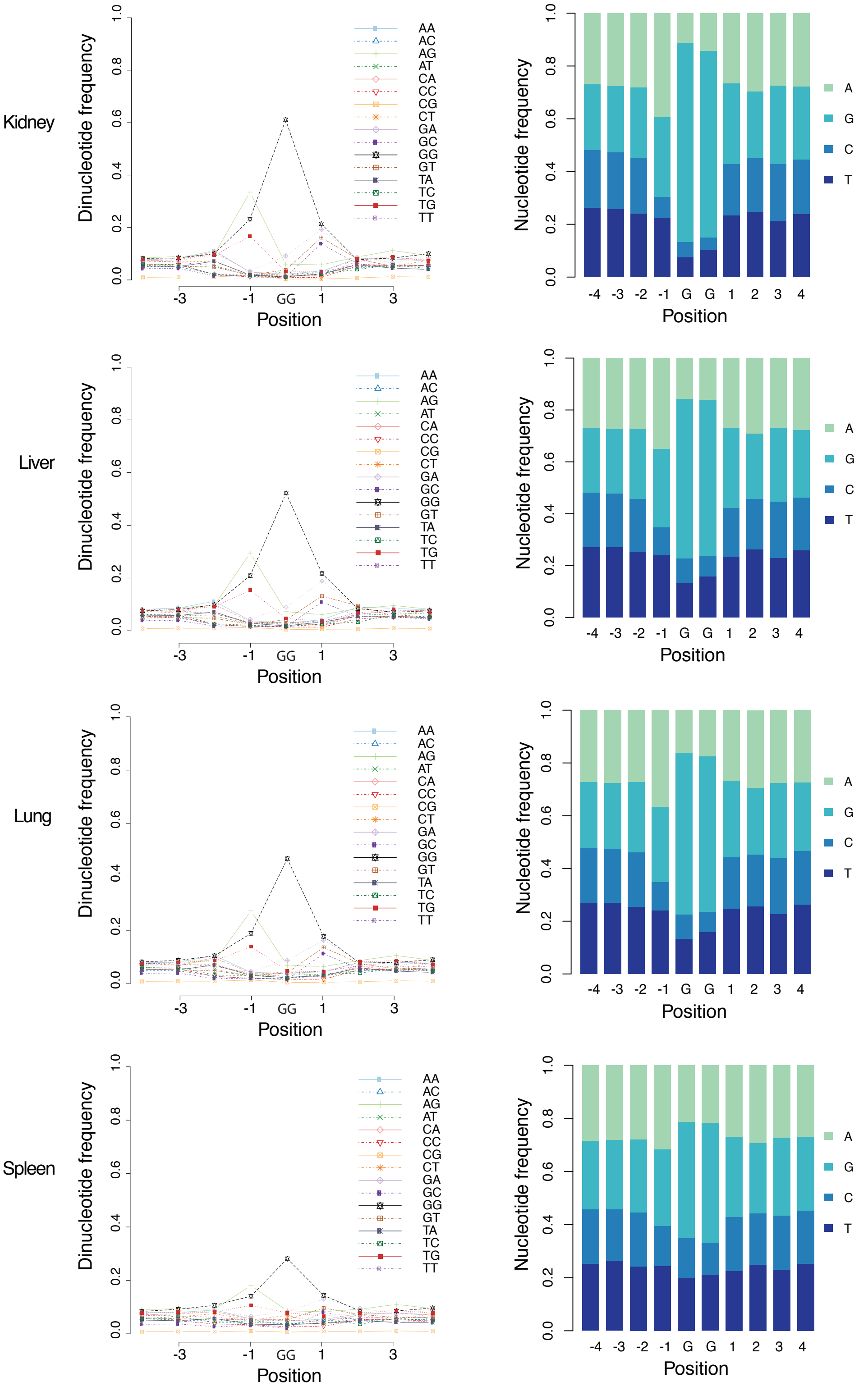

Supplementary Figure 3. Genome-wide dinucleotide frequency of cisplatin-induced DNA damage by Damage-seq. We observe a preference for adenine (A) 5’ to the GG dinucleotides at the damage site by Damage-seq (i.e., an enrichment of AGG, potentially due to antibody bias).

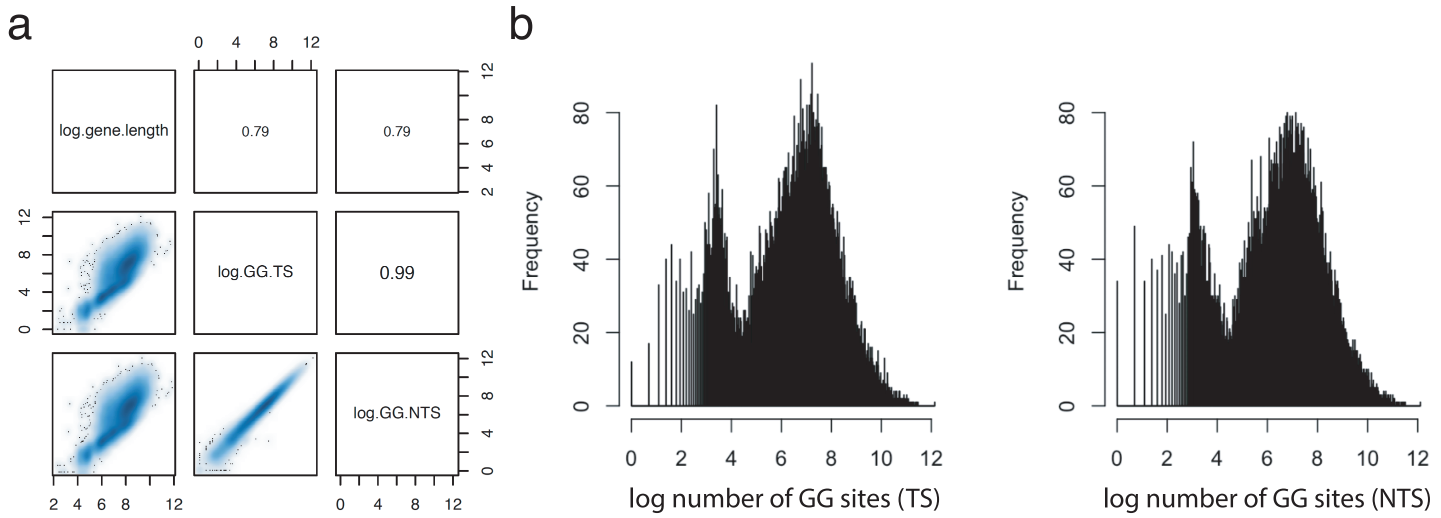

Supplementary Figure 4. GG dinucleotides in the transcribed and non-transcribed strands. a The number of GG dinucleotides in the transcribed strand is highly correlated with that in the non-transcribed strand. Gene length is not a good proxy for the number of GG dinucleotides. **b** Distribution of GG dinucleotides across all genes.

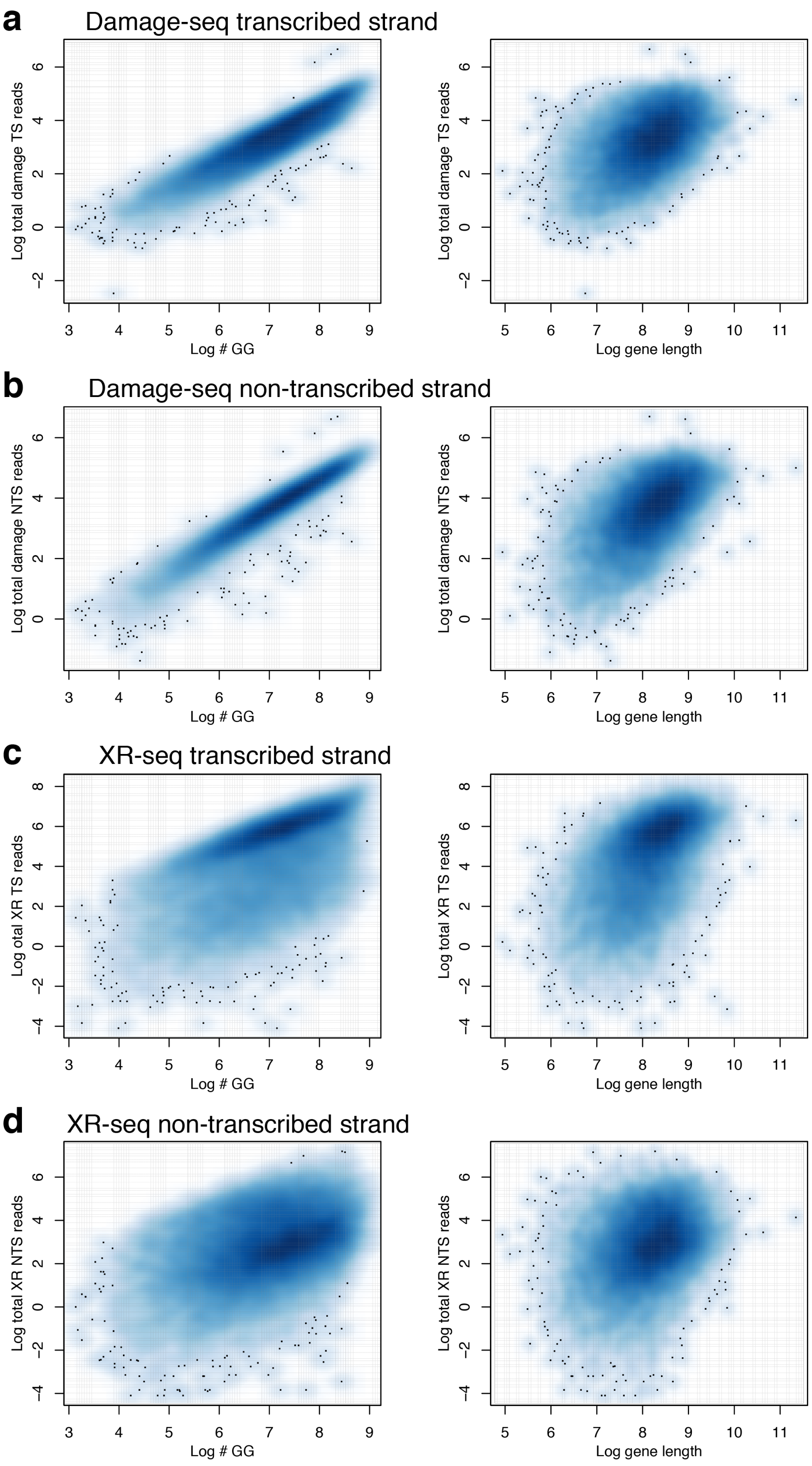

Supplementary Figure 5. Correlation of GG dinucleotides and gene length with DNA damage and excision repair. Scatter plot of the total number of reads versus the number of GG dinucleotides (left panel) and the gene lengths (right panel) in **a** Damage-seq in transcribed strand; **b** Damage-seq in non-transcribed strand; **c** XR-seq in transcribed strand, and **d** XR-seq in non-transcribed strand. Number of GG dinucleotides is used in normalization instead of gene length. Each dot corresponds to a gene.

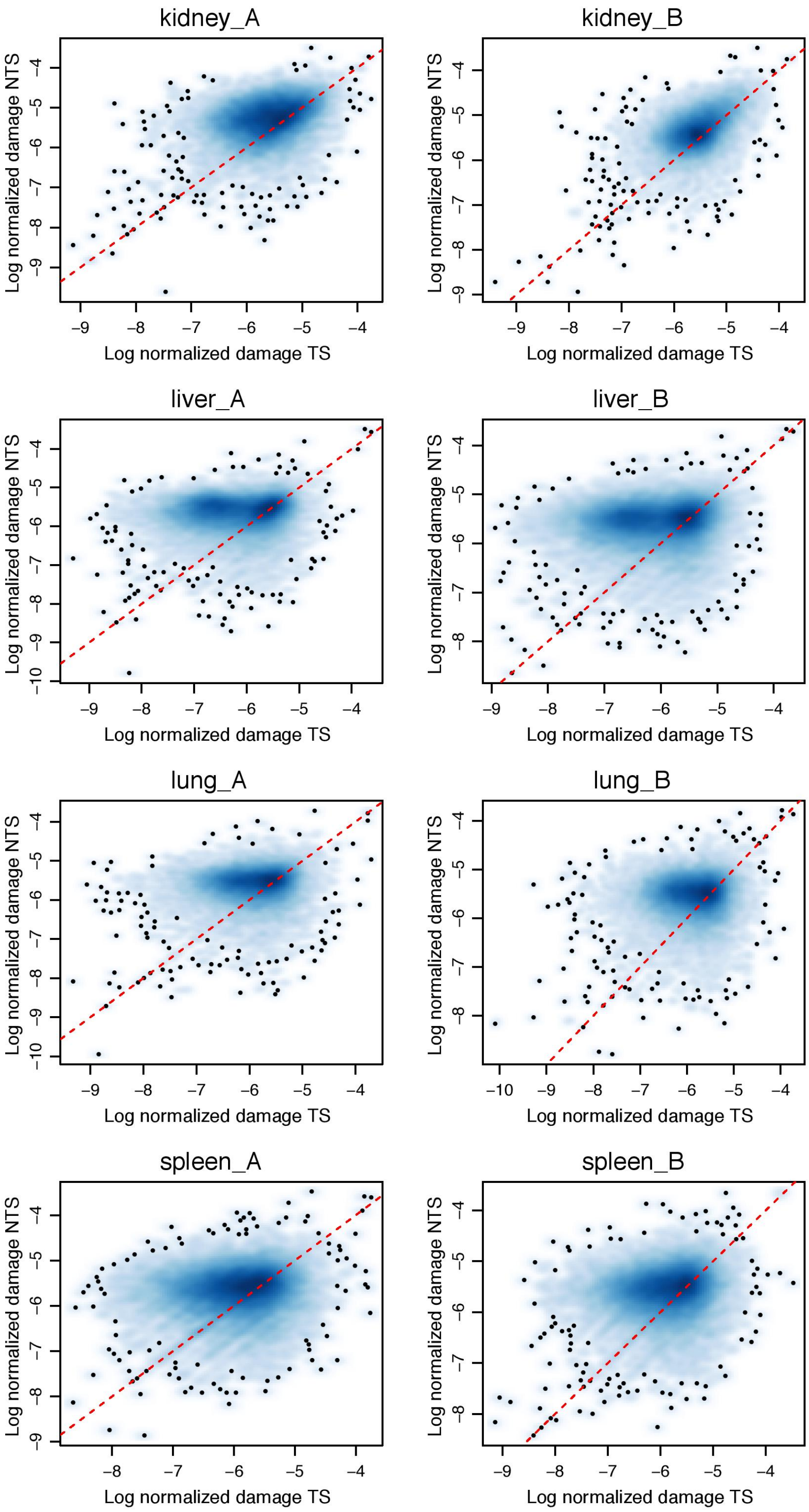

Supplementary Figure 6. Comparison of damage level between transcribed and non-transcribed strands. On the genome-wide scale, the damage level in the NTS strand is higher than that in TS, due to transcription-coupled repair in the TS. Each dot corresponds to a gene.

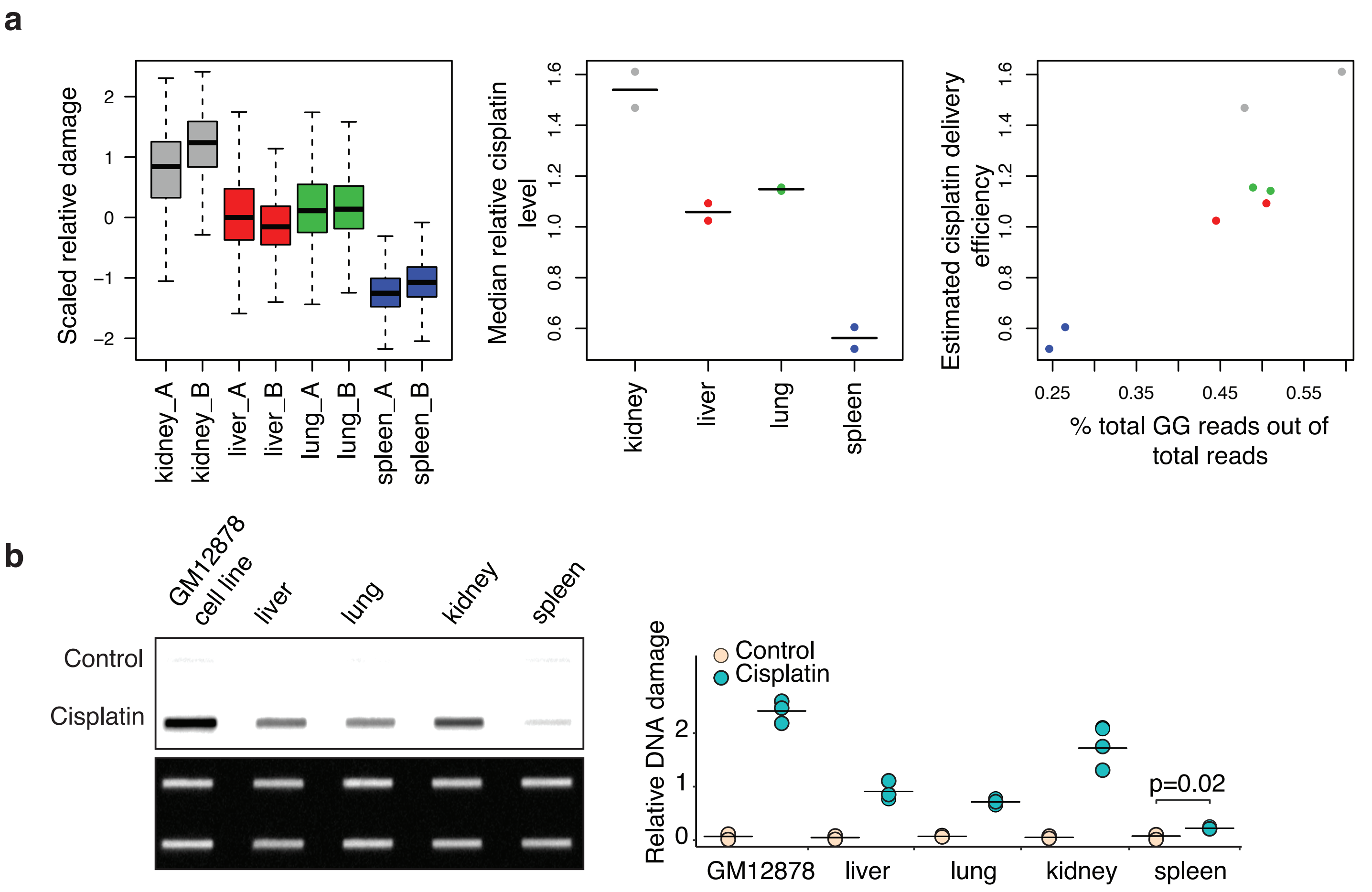

Supplementary Figure 7. Cisplatin delivery efficiency across organs. a *In silico* inference based on Damage-seq. Each sample is compared to a pseudo-reference sample to calculate a gene-specific relative damage (left panel). The median across all genes is taken and is used as an estimate for the overall damage level (middle panel). The relative damage level is highly correlated with the percentage of reads with GG dinucleotide 1-2 bp upstream of the 5' end out of the total number of reads (right panel), which is a proxy for the damage formation efficiency. **b** Immuno-slot blot analysis cisplatin-induced DNA-adducts. C57BL/6 wild-type mice were treated with cisplatin (10mg/Kg body weight) for four hours and the sacrificed. Kidney, liver, lung and spleen tissues were collected for analysis. Genomic DNA was isolated and probed for levels of cisplatin-DNA adducts with an α-Pt-(GpG) antibody (**b**, left top panel) in a slot-blot experiment. Anti-DNA antibody (**b**, bottom panel) was used as an internal control. (**b**, right panel), Cisplatin-DNA adduct repair was quantified from experiments performed as in (**b**, left panel). Cisplatin damaged DNA from GM12878 cell line was loaded as positive control. Experiments were performed with three biological replicates.

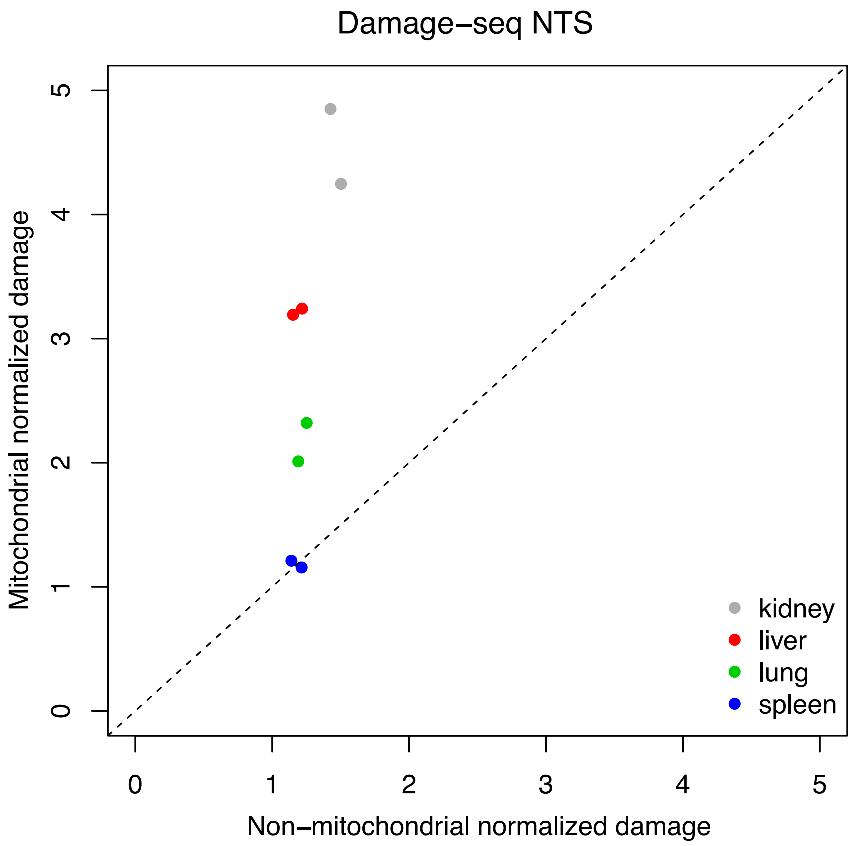

Supplementary Figure 8. Mitochondrial DNA damage. Normalized damage from Damage-seq is plotted for the nuclear DNA (x-axis) and the mitochondrial DNA (y-axis). Mitochondrial DNA is a major target of cisplatin damage, potentially due to the many copies of mitochondria and mitochondrial DNA.

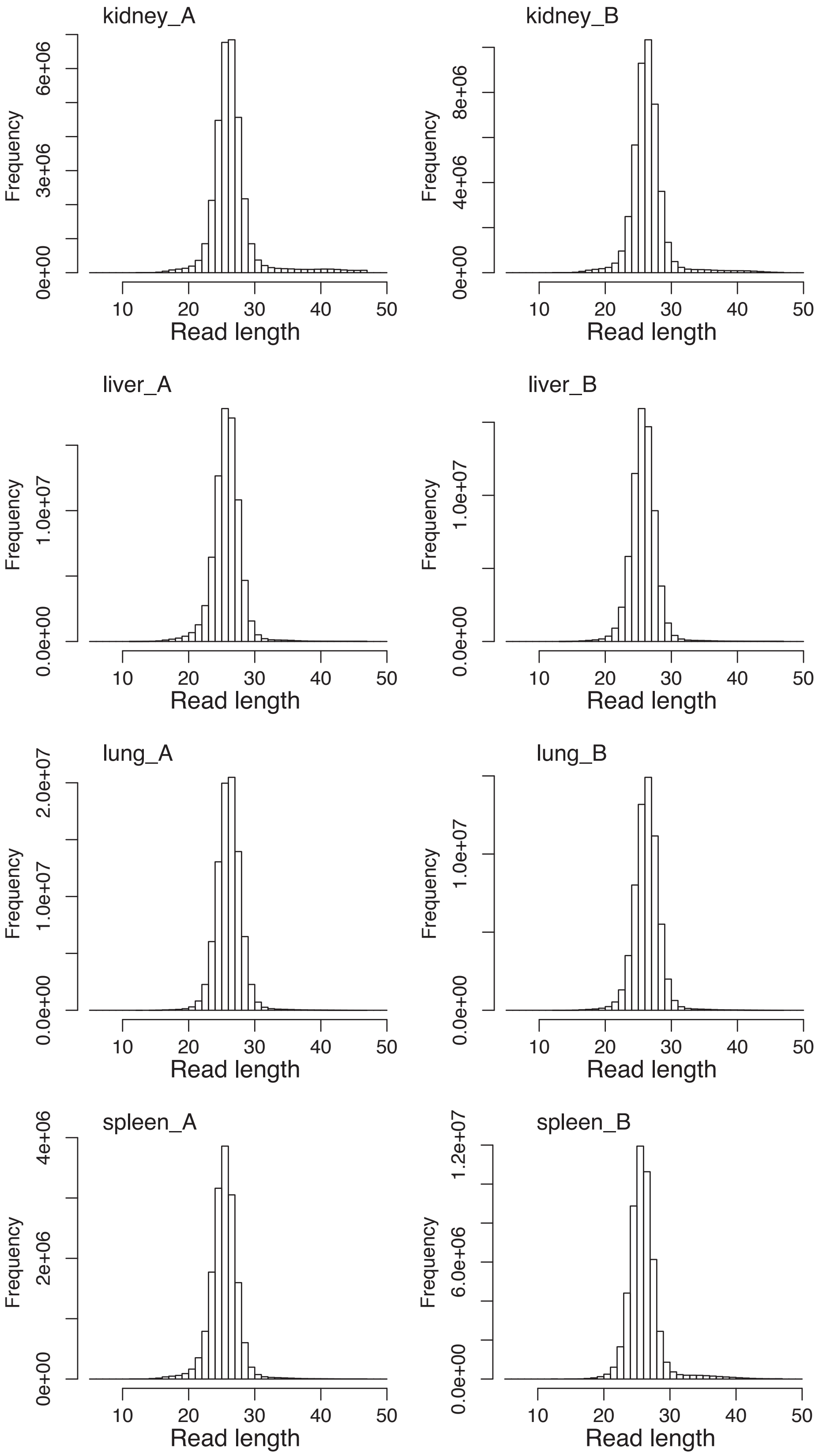

Supplementary Figure 9. Read length distribution for XR-seq after adaptor trimming. Lengths of the excised oligomers fall within the range of 21 to 31 bp across all samples with peak at 26-27nt.

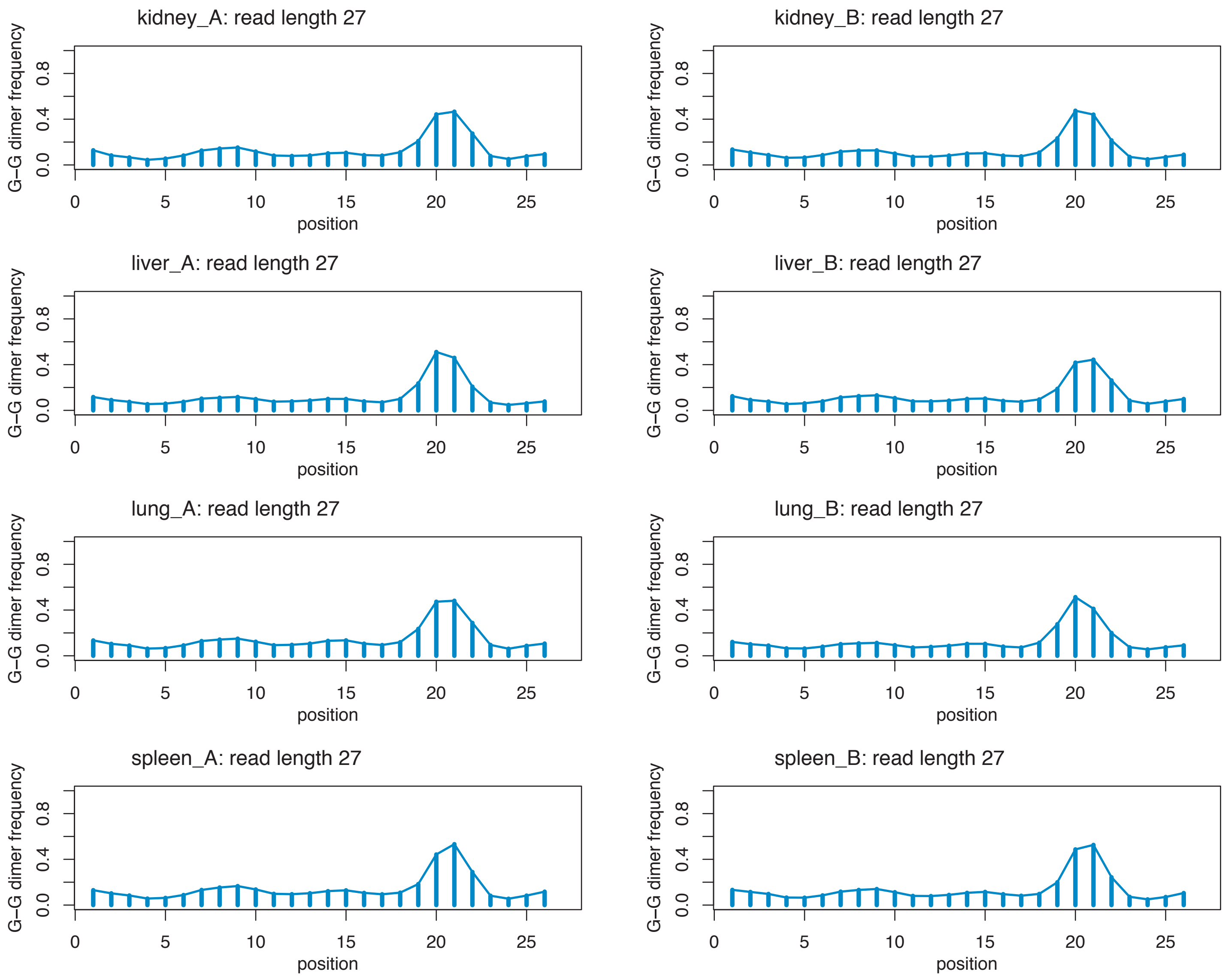

Supplementary Figure 10. GG dinucleotide enrichment in XR-seq. GG dinucleotide is enriched 5-8 bp upstream of the 3' end of the reads across all samples. Only reads with length 27 bp are shown. Same results are observed across all read lengths.

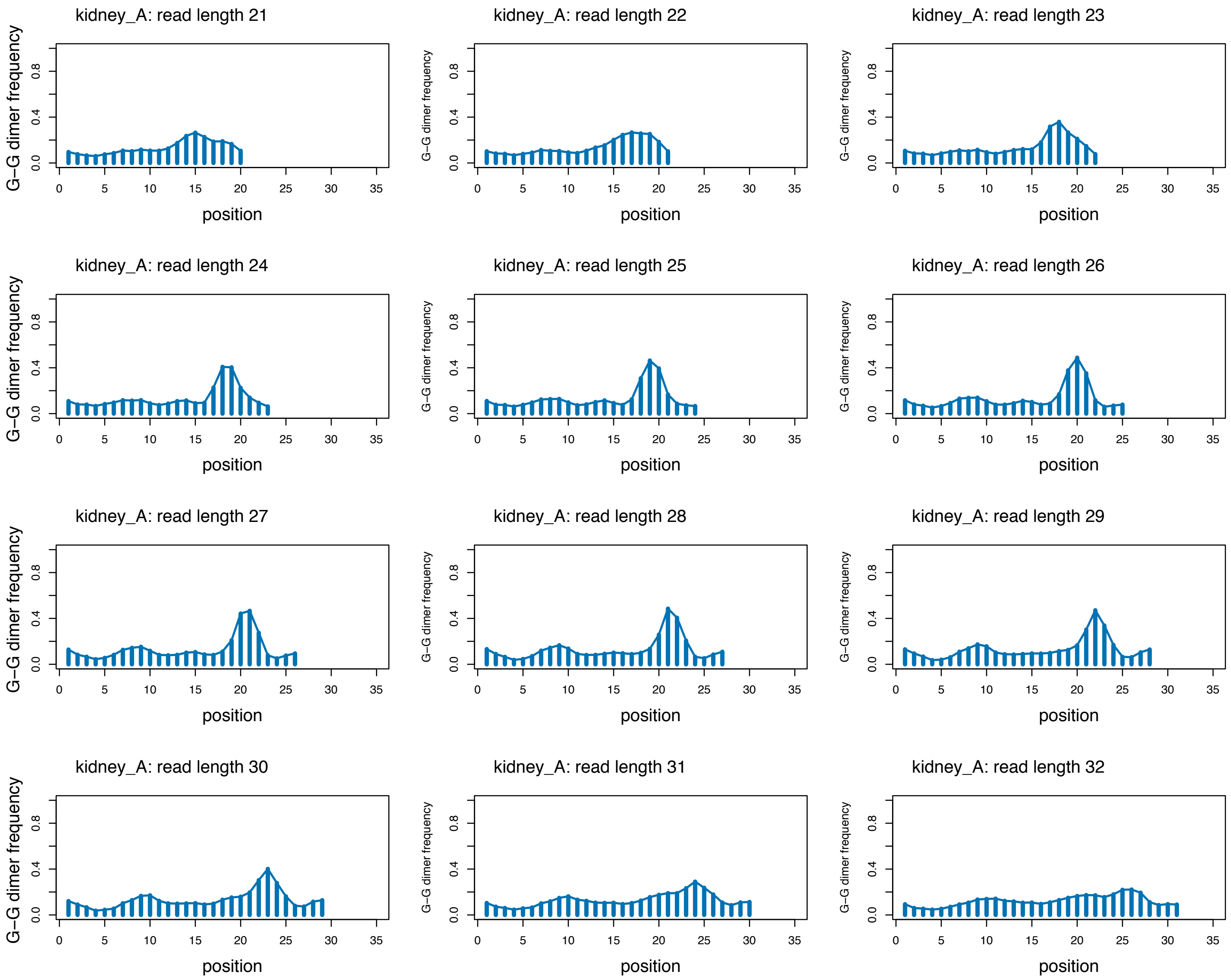

Supplementary Figure 11. GG dinucleotide enrichment in XR-seq across different read lengths. GG dinucleotide is enriched 5-8 bp upstream of the 3' end of the reads. Only the first kidney sample is shown. Same results are observed in all samples and organs.

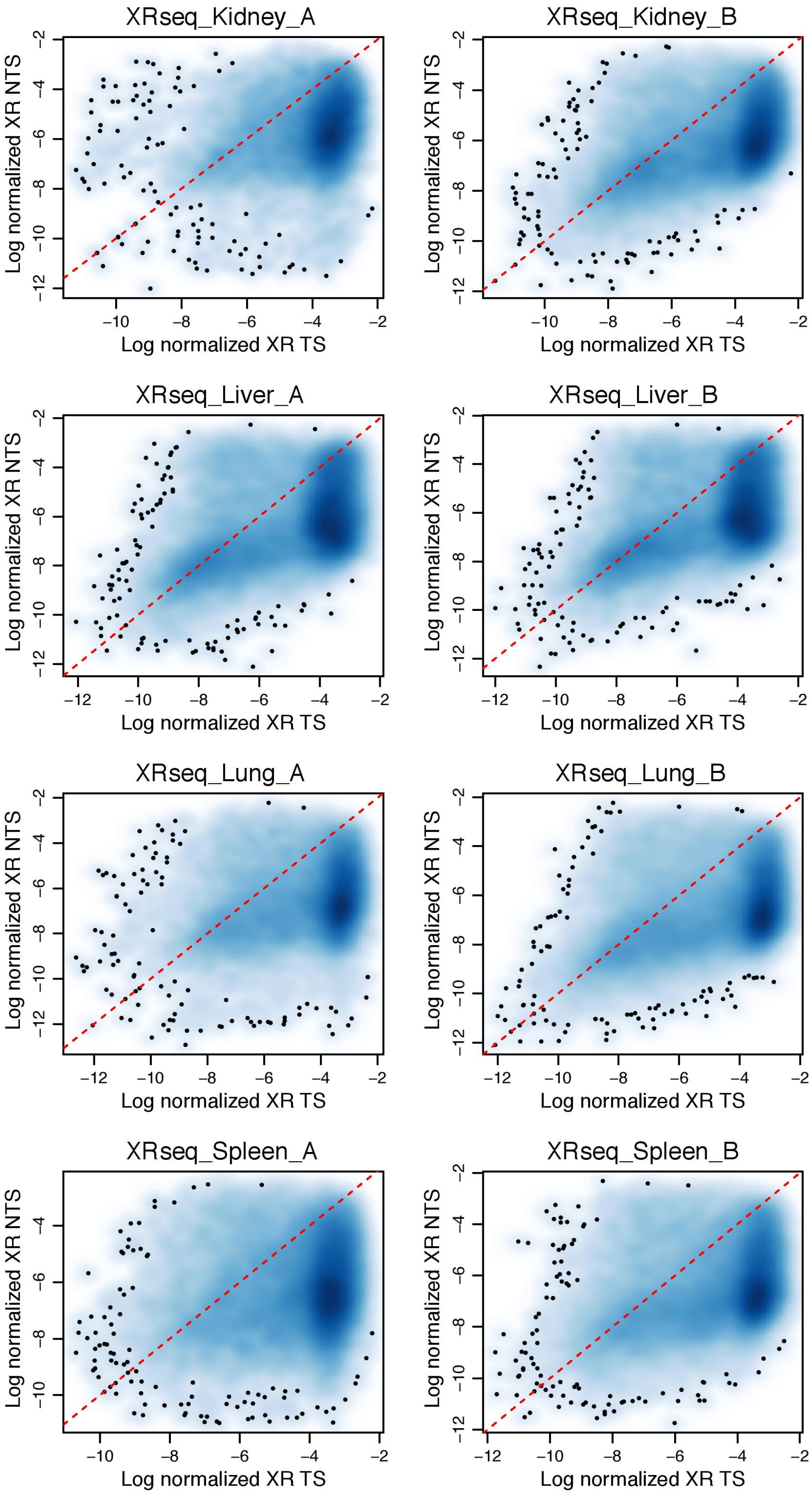

Supplementary Figure 12. Excision repair in transcribed versus non-transcribed strand. On the genome-wide scale, excision repair in TS is higher than that in NTS, due to transcription-coupled repair in the TS. Each dot corresponds to a gene.

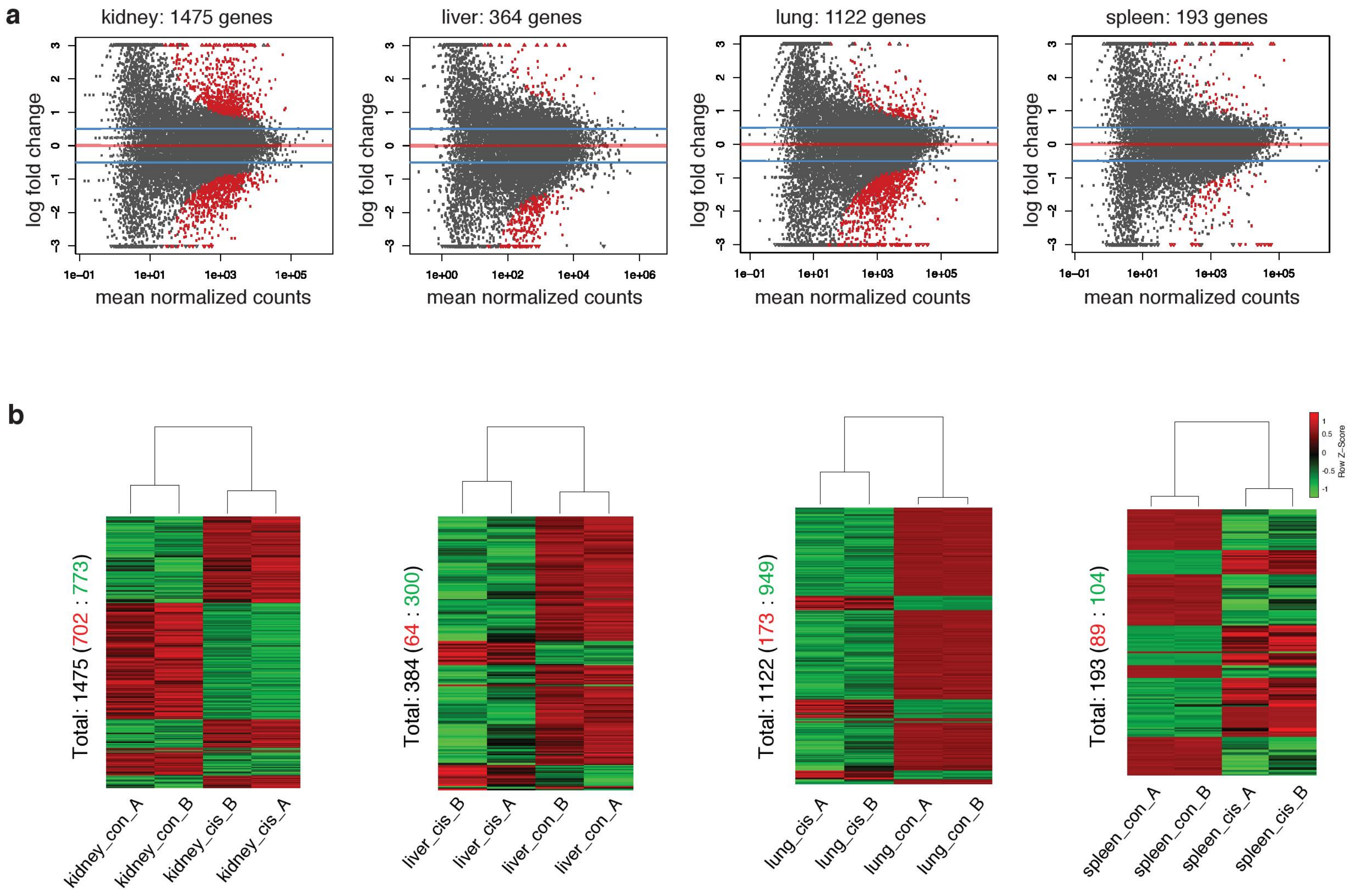

Supplementary Figure 13. Differential gene expression between cisplatin treated group and control group. a Results from the DESeq2 analysis with genes being up- and down-regulated. **b** Heatmaps of significantly up- and down-regulated genes. Red color indicates up-regulated genes in cisplatin treated group compared to control group, whereas green color indicates down-regulated genes.

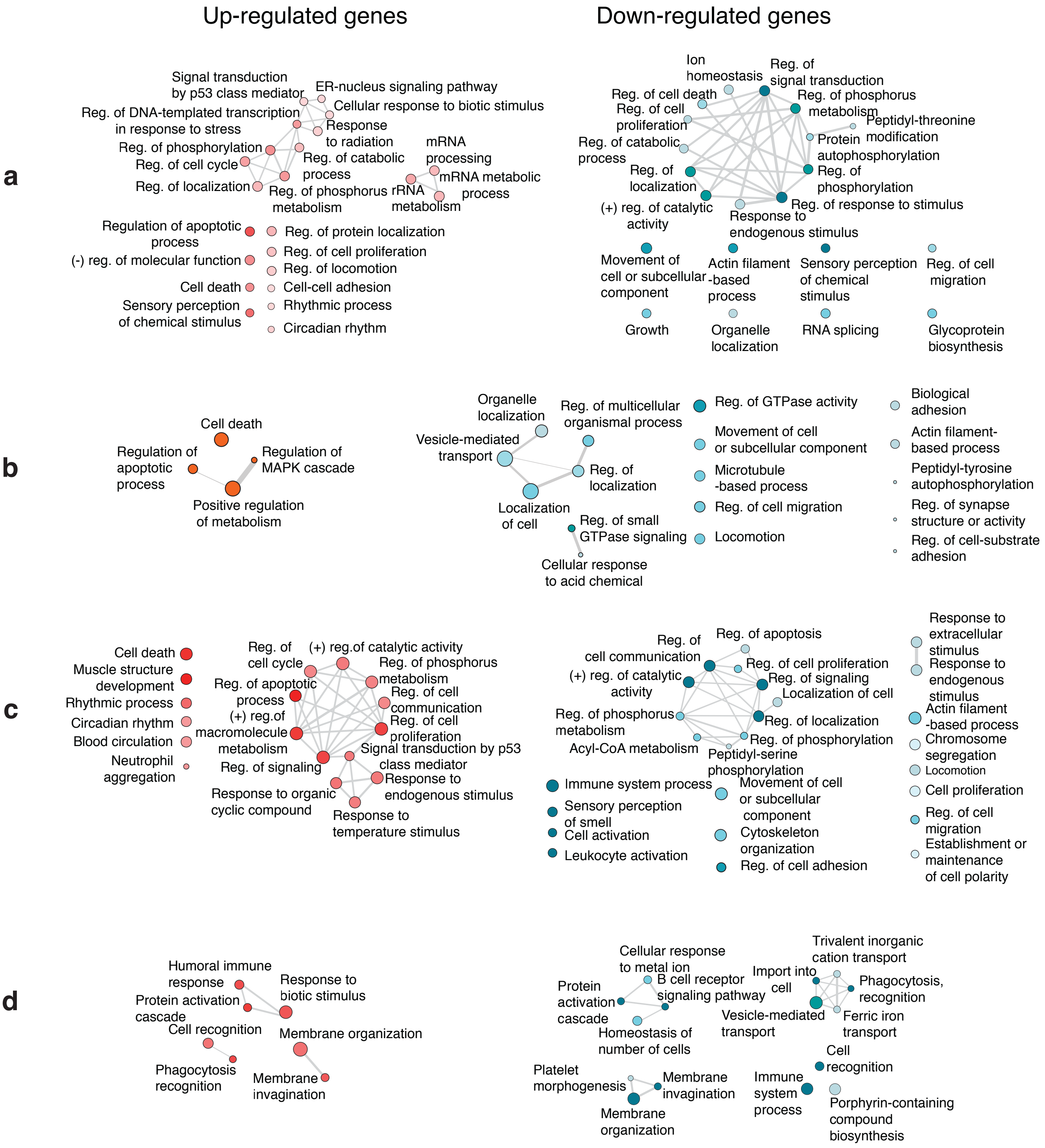
Up-regulated genes
Down-regulated genes
a
Signal transduction by p53 class mediator
ER-nucleus signaling pathway
Reg. of DNA-templated transcription in response to stress
Cellular response to biotic stimulus
Response to radiation
Reg. of phosphorylation
Reg. of cell cycle
Reg. of catabolic process
Reg. of localization
Reg. of phosphorus metabolism
mRNA processing
mRNA metabolic process
rRNA metabolism
Regulation of apoptotic process
(-) reg. of molecular function
Cell death
Sensory perception of chemical stimulus
Reg. of protein localization
Reg. of cell proliferation
Reg. of locomotion
Cell-cell adhesion
Rhythmic process
Circadian rhythm
Ion homeostasis
Reg. of signal transduction
Reg. of cell death
Reg. of phosphorus metabolism
Reg. of cell proliferation
Peptidyl-threonine modification
Reg. of catabolic process
Protein autophosphorylation
Reg. of localization
Reg. of phosphorylation
(+) reg. of catalytic activity
Response to endogenous stimulus
Reg. of response to stimulus
Movement of cell or subcellular component
Actin filament-based process
Sensory perception of chemical stimulus
Reg. of cell migration
Growth
Organelle localization
RNA splicing
Glycoprotein biosynthesis
b
Cell death
Regulation of apoptotic process
Regulation of MAPK cascade
Positive regulation of metabolism
Organelle localization
Reg. of multicellular organismal process
Vesicle-mediated transport
Reg. of localization
Localization of cell
Reg. of small GTPase signaling
Cellular response to acid chemical
Reg. of GTPase activity
Movement of cell or subcellular component
Microtubule-based process
Reg. of cell migration
Locomotion
Biological adhesion
Actin filament-based process
Peptidyl-tyrosine autophosphorylation
Reg. of synapse structure or activity
Reg. of cell-substrate adhesion
c
Cell death
Muscle structure development
Rhythmic process
Circadian rhythm
Blood circulation
Neutrophil aggregation
Reg. of cell cycle
(+) reg.of catalytic activity
Reg. of phosphorus metabolism
Reg. of apoptotic process
Reg. of cell communication
(+) reg.of macromolecule metabolism
Reg. of cell proliferation
Signal transduction by p53 class mediator
Reg. of signaling
Response to endogenous stimulus
Response to organic cyclic compound
Response to temperature stimulus
Reg. of cell communication
Reg. of apoptosis
Reg. of cell proliferation
(+) reg. of catalytic activity
Reg. of signaling
Localization of cell
Reg. of phosphorus metabolism
Reg. of localization
Reg. of phosphorylation
Acyl-CoA metabolism
Peptidyl-serine phosphorylation
Response to extracellular stimulus
Response to endogenous stimulus
Actin filament-based process
Chromosome segregation
Locomotion
Cell proliferation
Reg. of cell migration
Establishment or maintenance of cell polarity
Immune system process
Sensory perception of smell
Cell activation
Leukocyte activation
Movement of cell or subcellular component
Cytoskeleton organization
Reg. of cell adhesion
d
Humoral immune response
Response to biotic stimulus
Protein activation cascade
Cell recognition
Membrane organization
Phagocytosis recognition
Membrane invagination
Cellular response to metal ion
B cell receptor signaling pathway
Protein activation cascade
Homeostasis of number of cells
Trivalent inorganic cation transport
Import into cell
Phagocytosis, recognition
Vesicle-mediated transport
Ferric iron transport
Platelet morphogenesis
Membrane invagination
Membrane organization
Cell recognition
Immune system process
Porphyrin-containing compound biosynthesis

Supplementary Figure 14. Gene Ontology (GO) term enrichment networks. GO term enrichment networks for mRNAs with increased abundance (left, node color from dark red to light red) and mRNAs with decreased abundance (right, node color from dark blue to light blue). GO analysis was performed using the GO term enrichment analysis (http://geneontology.org/) using a false discovery rate (FDR) cut-off of 0.05, and applying Fisher's Exact with FDR multiple test correction, querying biological process enrichment for each gene set. GO term enrichment results were further processed with REViGO using the "Tiny (0.4)" term similarity filter and SimRel score as the semantic similarity measure. Resulted GO term network on REViGO was loaded into Cytoscape v3.4.0 for visualization. Nodes representing terms with a frequency greater than 1 % in the REViGO output were eliminated for being too general, and only edge interactions with a score > 0.25 were represented in the networks. Nodes are shaded according to *p*-values (darkest red indicating the smallest *p*-value). Node size indicates the frequency at which a given GO term is found in the *Mus. musculus* genome. Edges connect highly similar GO terms and edge thickness represents degree of similarity. GO term enrichment networks for mRNAs that were differentially expressed in mouse **a** kidney. **b** liver. **c** lung. **d** spleen.

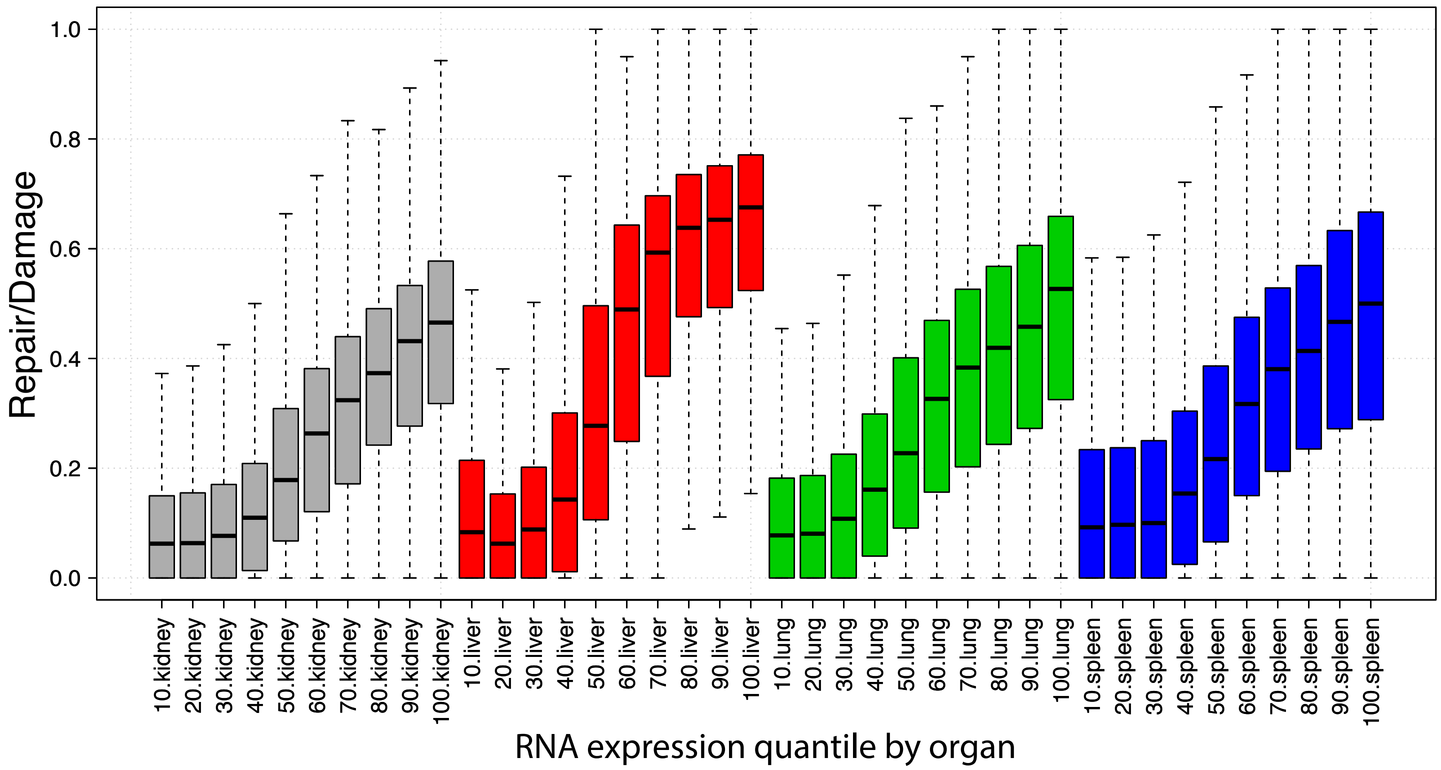

Supplementary Figure 15. Relationship between gene expression and repair efficiency. Genes are grouped according to their gene expression quantiles in each organ. The vertical axis, repair/damage, quantifies the proportion of overall accumulated transcription-coupled repair (measured using the TS) over the total damage induced (measured using the NTS). Liver has the highest ratio, i.e., repair efficiency.

a

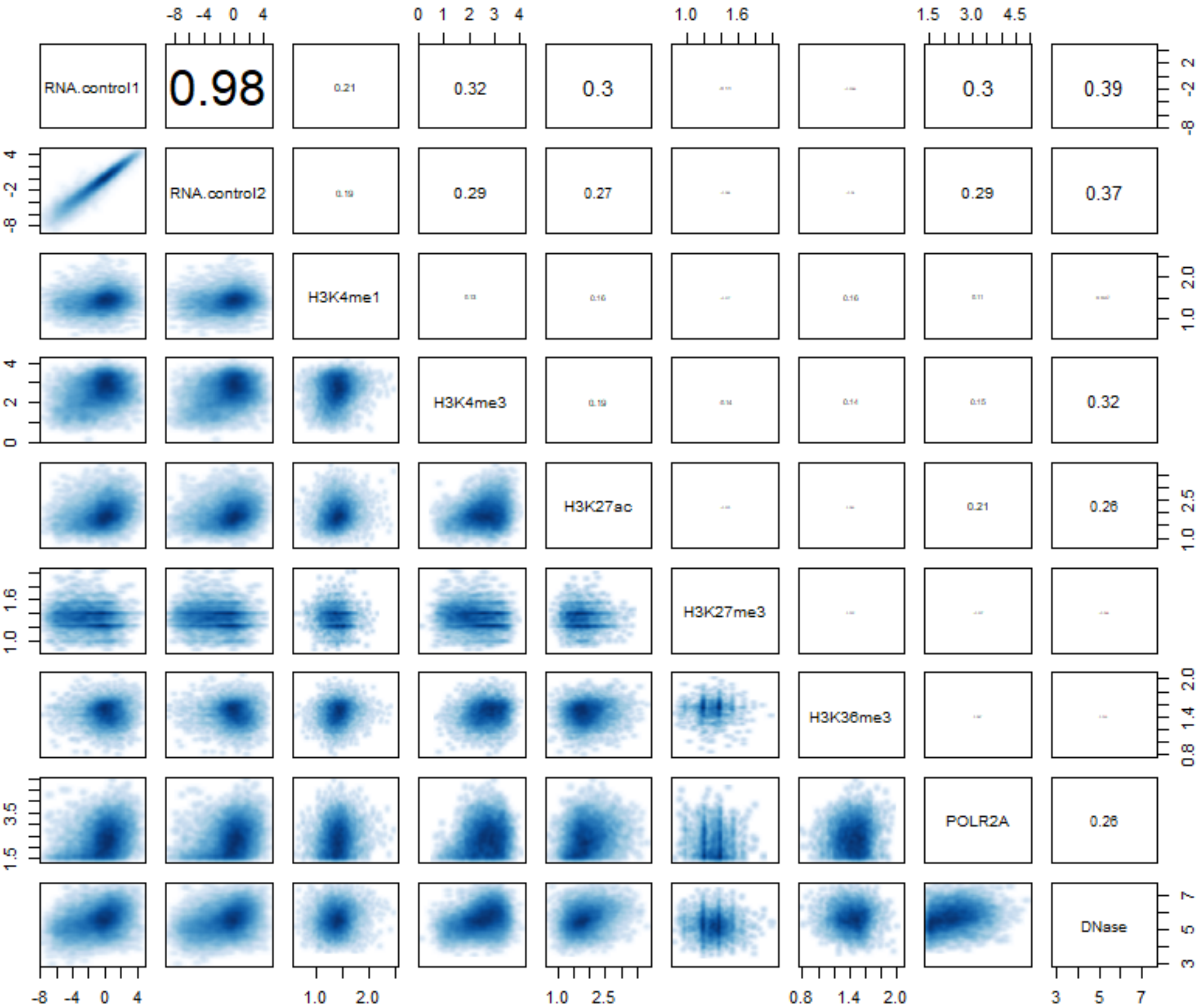
RNA.control1
RNA.control2
H3K4me1
H3K4me3
H3K27ac
H3K27me3
H3K36me3
POLR2A
DNase
0.98
0.21
0.32
0.3
0.3
0.39
0.29
0.27
0.29
0.37
0.32
0.21
0.26
0.26

b

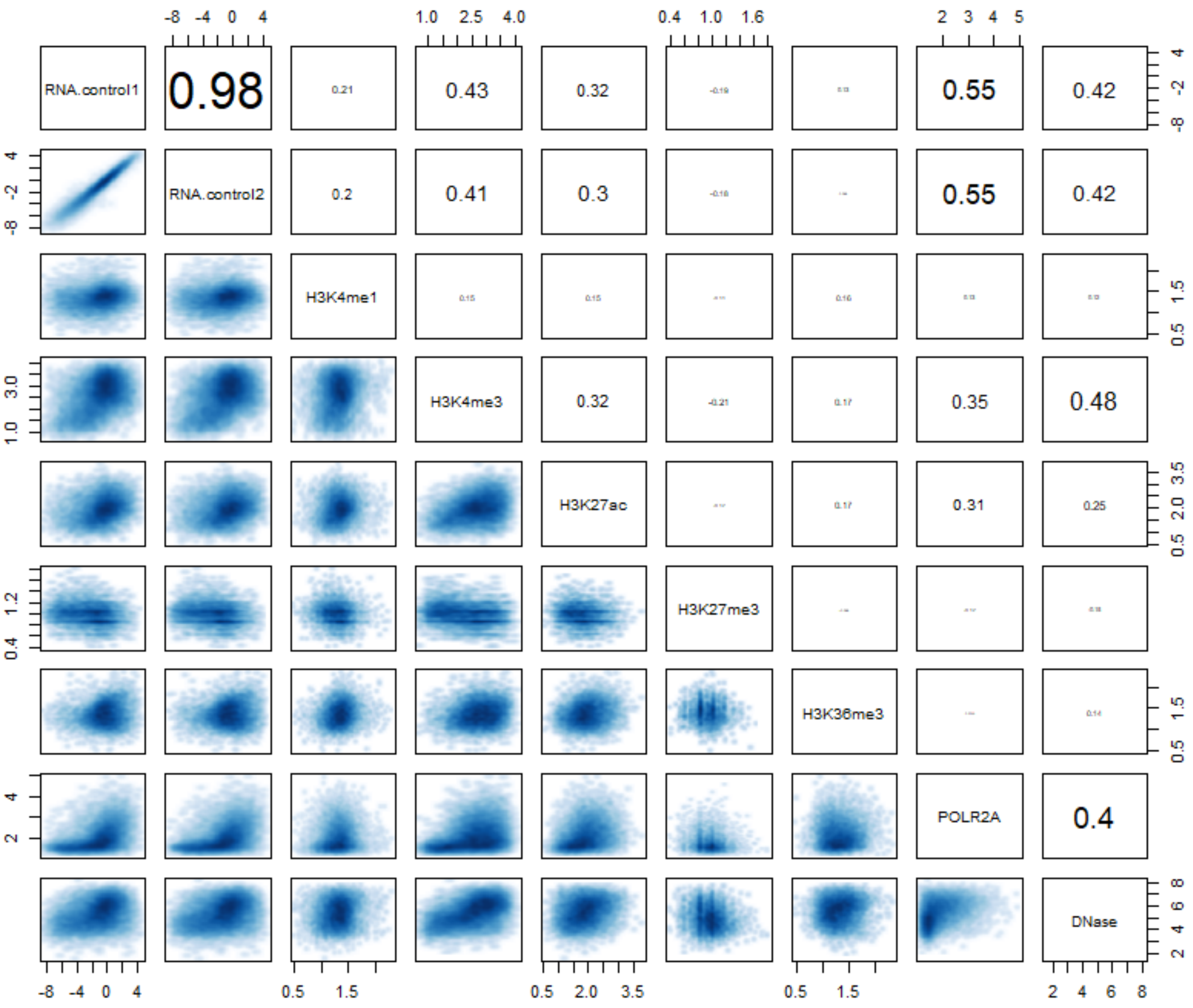
RNA.control1
RNA.control2
H3K4me1
H3K4me3
H3K27ac
H3K27me3
H3K36me3
POLR2A
DNase
0.98
0.21
0.43
0.32
0.55
0.42
0.2
0.41
0.3
0.55
0.42
0.32
0.35
0.48
0.31
0.25
0.4

c

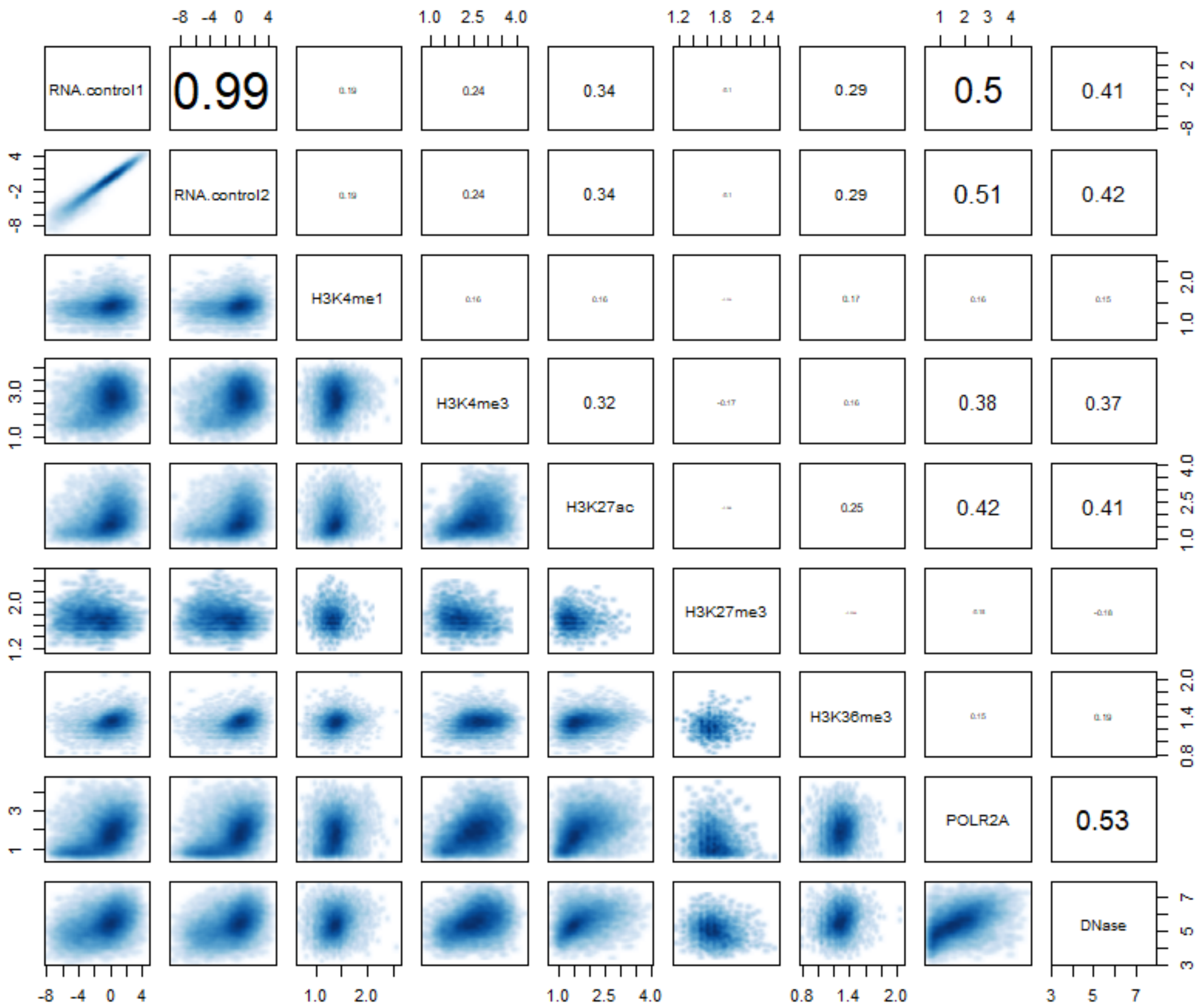
RNA.control1
RNA.control2
H3K4me1
H3K4me3
H3K27ac
H3K27me3
H3K36me3
POLR2A
DNase
0.99
0.24
0.34
0.29
0.5
0.41
0.24
0.34
0.29
0.51
0.42
0.32
0.38
0.37
0.25
0.42
0.41
0.53

d

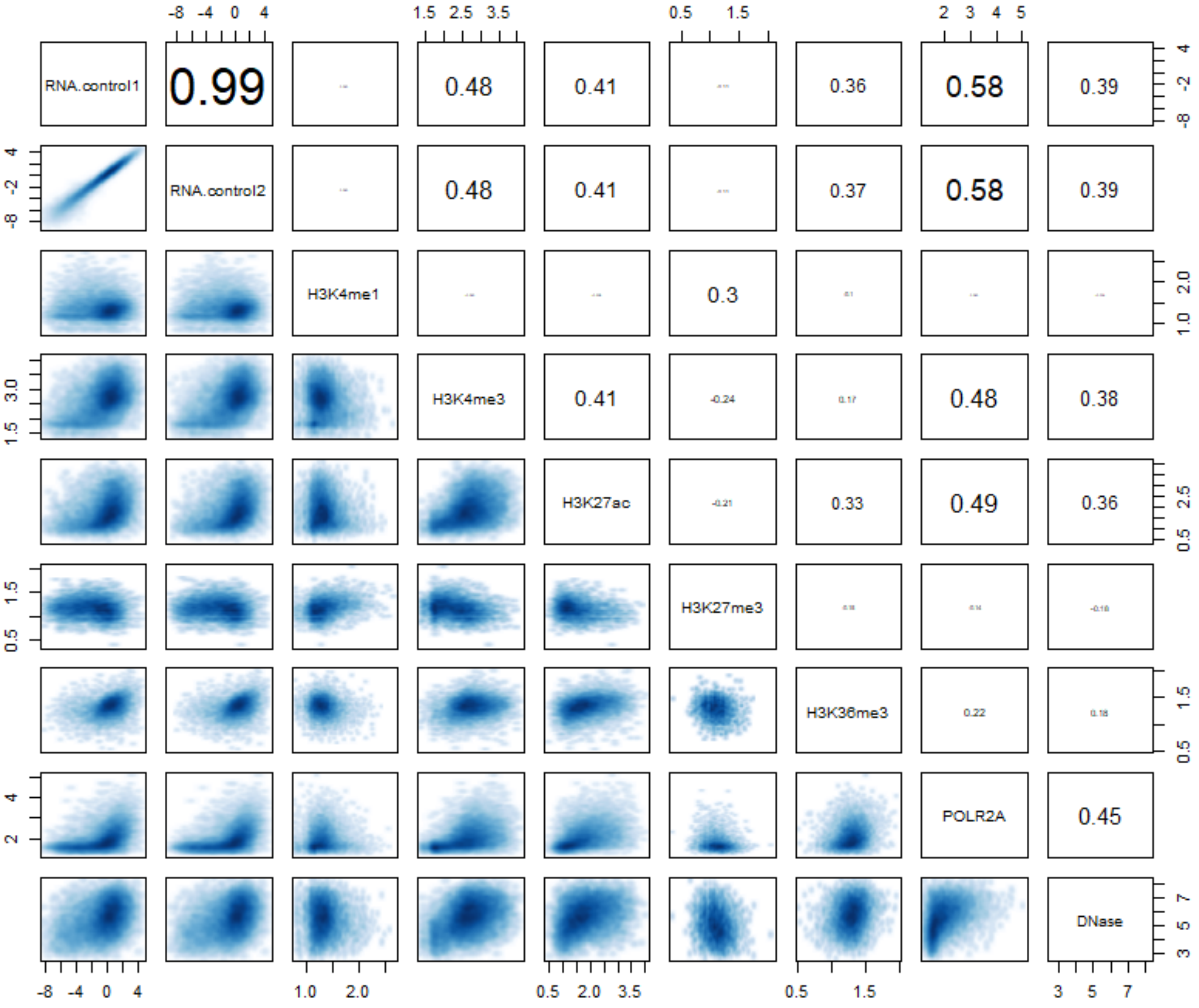

Supplementary Figure 16. Relationship between gene expression and epigenomic profiles in control samples. Pairwise scatterplots are shown, with the lower panel being the smooth scatterplots and the upper panel being the Spearman correlation coefficient for each pair of gene-specific measurements. The two control samples from each organ has the highest correlation. H3K27me3 is slightly negatively correlated with gene expression, while all other epigenomic markers have positive correlation coefficients. **a** kidney. **b** liver. **c** lung. **d** spleen.

a

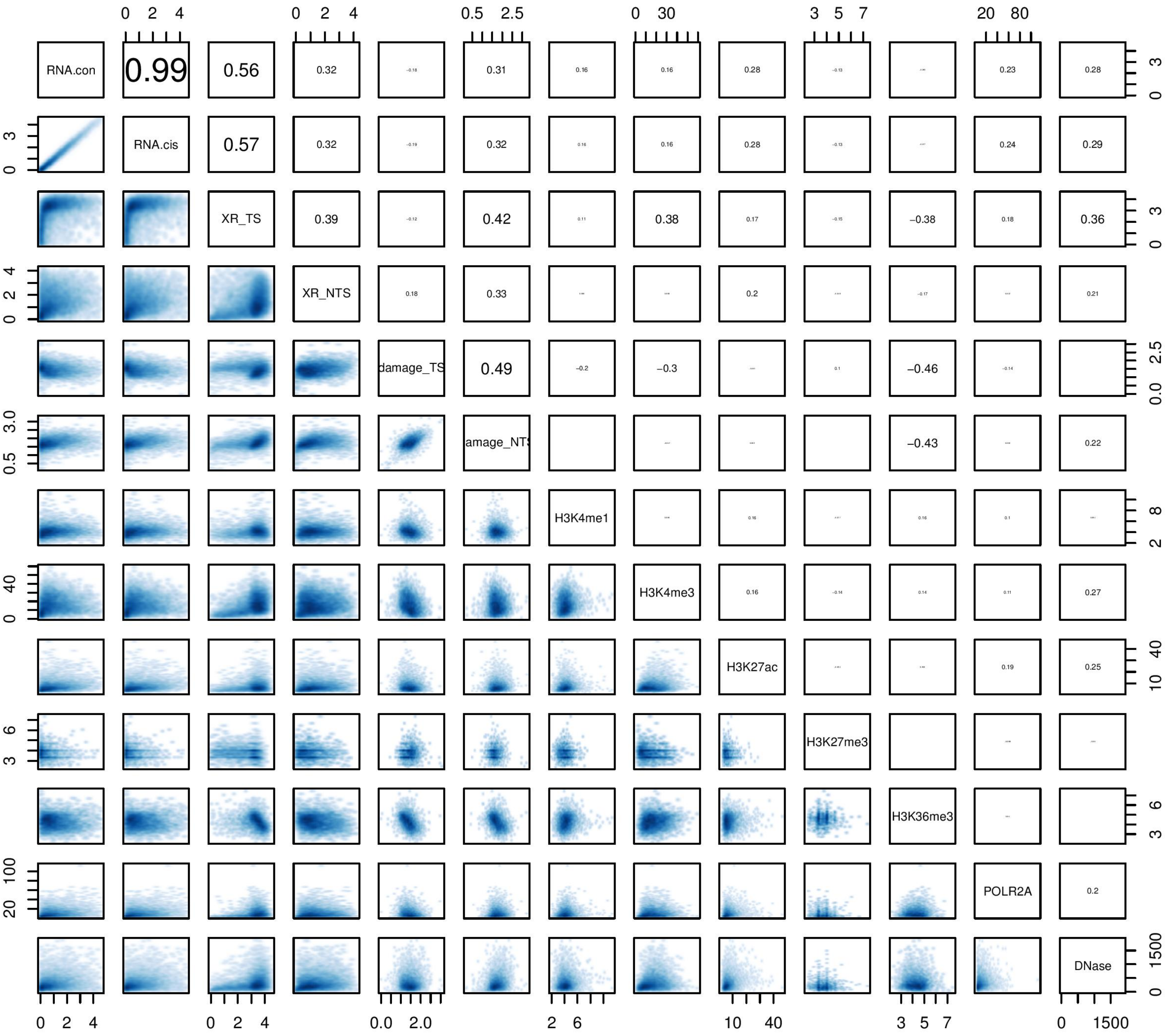
RNA.con
0.99
0.56
0.32
0.31
0.16
0.16
0.28
0.23
0.28
RNA.cis
0.57
0.32
0.32
0.16
0.28
0.24
0.29
XR_TS
0.39
0.42
0.38
0.17
−0.38
0.18
0.36
XR_NTS
0.18
0.33
0.2
0.21
damage_TS
0.49
−0.2
−0.3
−0.46
amage_NTS
−0.43
0.22
H3K4me1
H3K4me3
0.16
0.27
H3K27ac
0.19
0.25
H3K27me3
H3K36me3
POLR2A
0.2
DNase

b

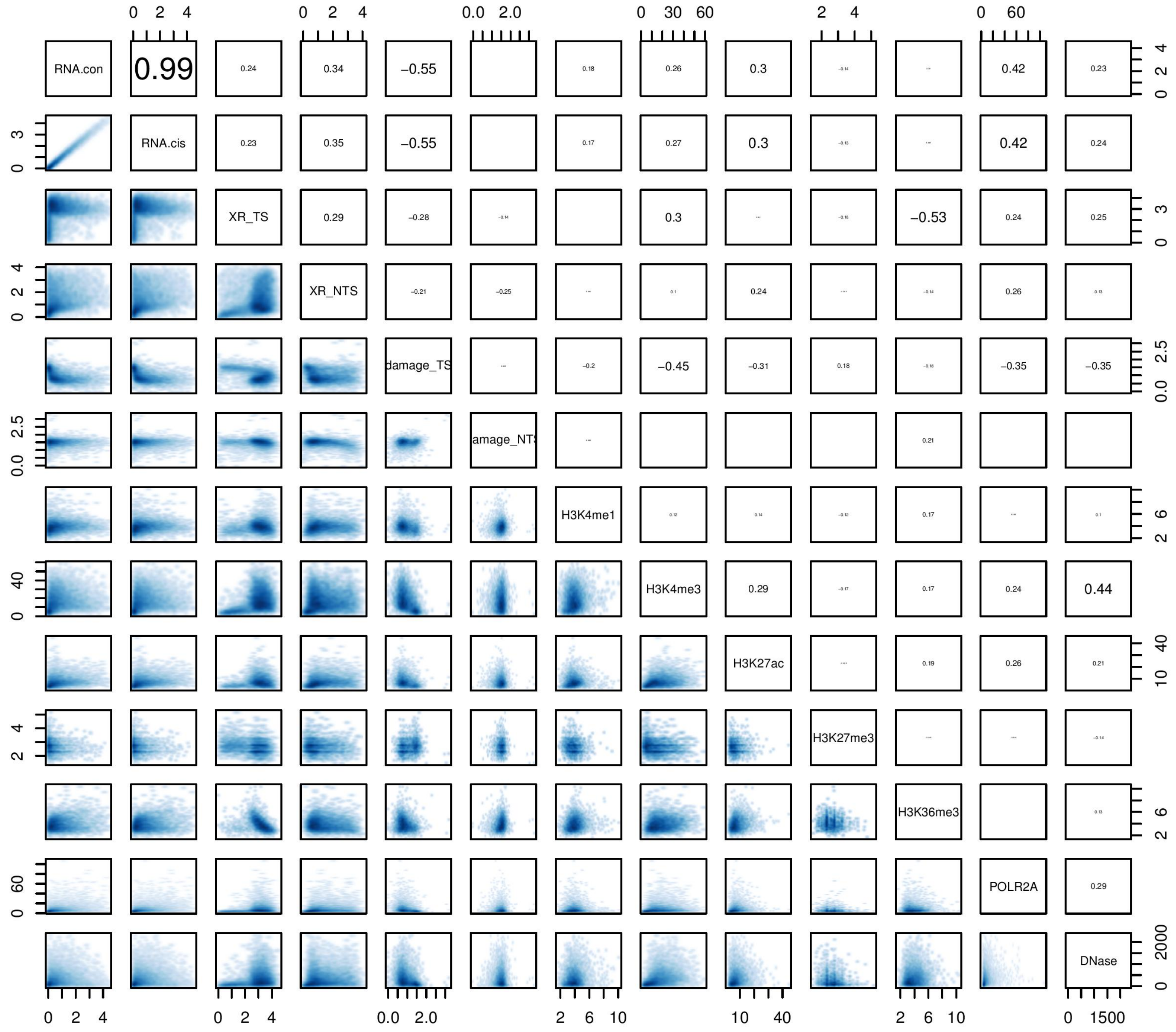

RNA.con
RNA.cis
XR_TS
XR_NTS
damage_TS
amage_NTS
H3K4me1
H3K4me3
H3K27ac
H3K27me3
H3K36me3
POLR2A
DNase
0.99
0.24
0.34
−0.55
0.18
0.26
0.3
0.42
0.23
0.23
0.35
−0.55
0.17
0.27
0.3
0.42
0.24
0.29
−0.28
0.3
−0.53
0.24
0.25
−0.21
−0.25
0.24
0.26
−0.2
−0.45
−0.31
0.18
−0.35
−0.35
0.21
0.17
0.29
0.17
0.24
0.44
0.19
0.26
0.21
0.29

c

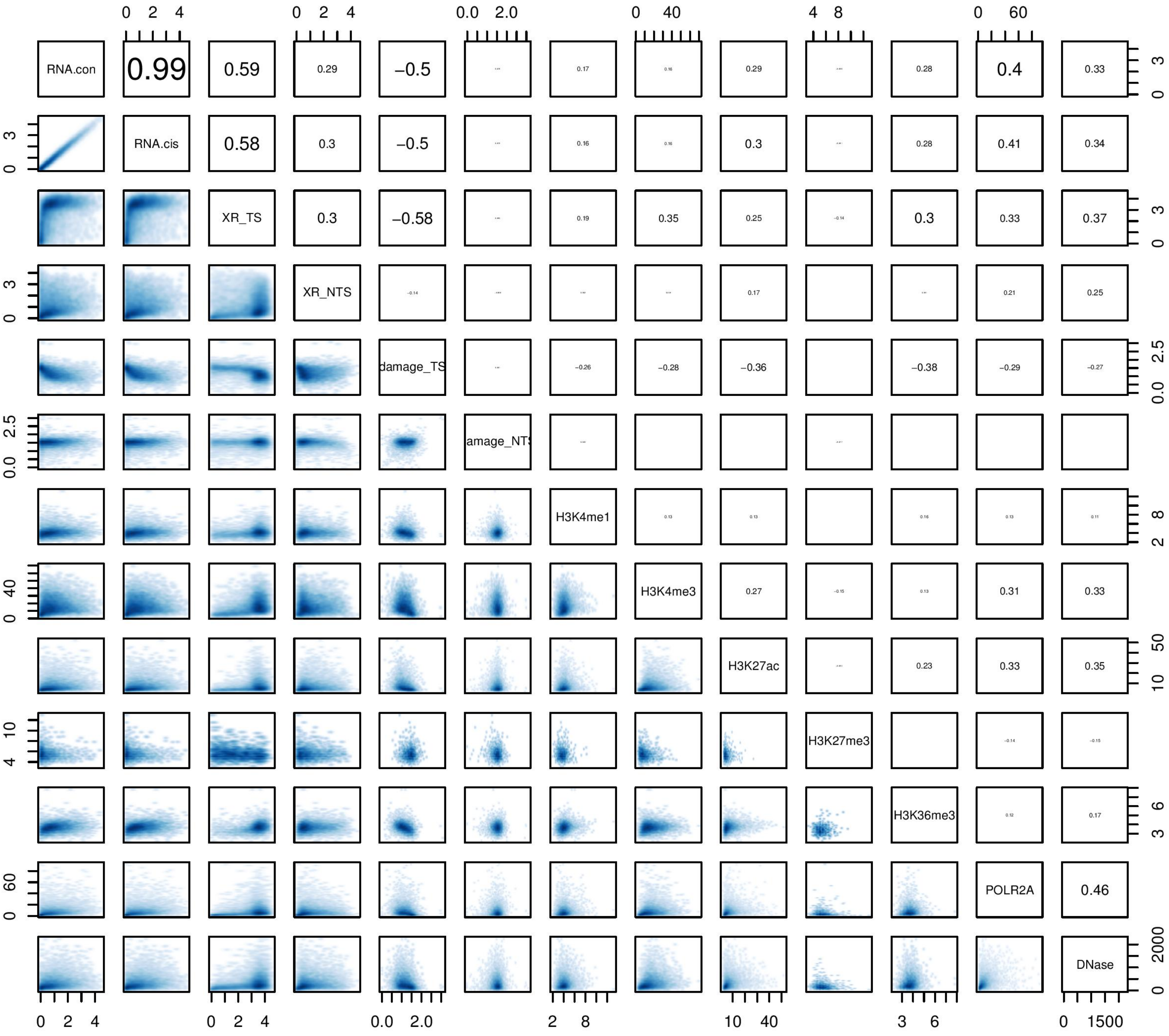
RNA.con
RNA.cis
XR_TS
XR_NTS
damage_TS
damage_NTS
H3K4me1
H3K4me3
H3K27ac
H3K27me3
H3K36me3
POLR2A
DNase

d

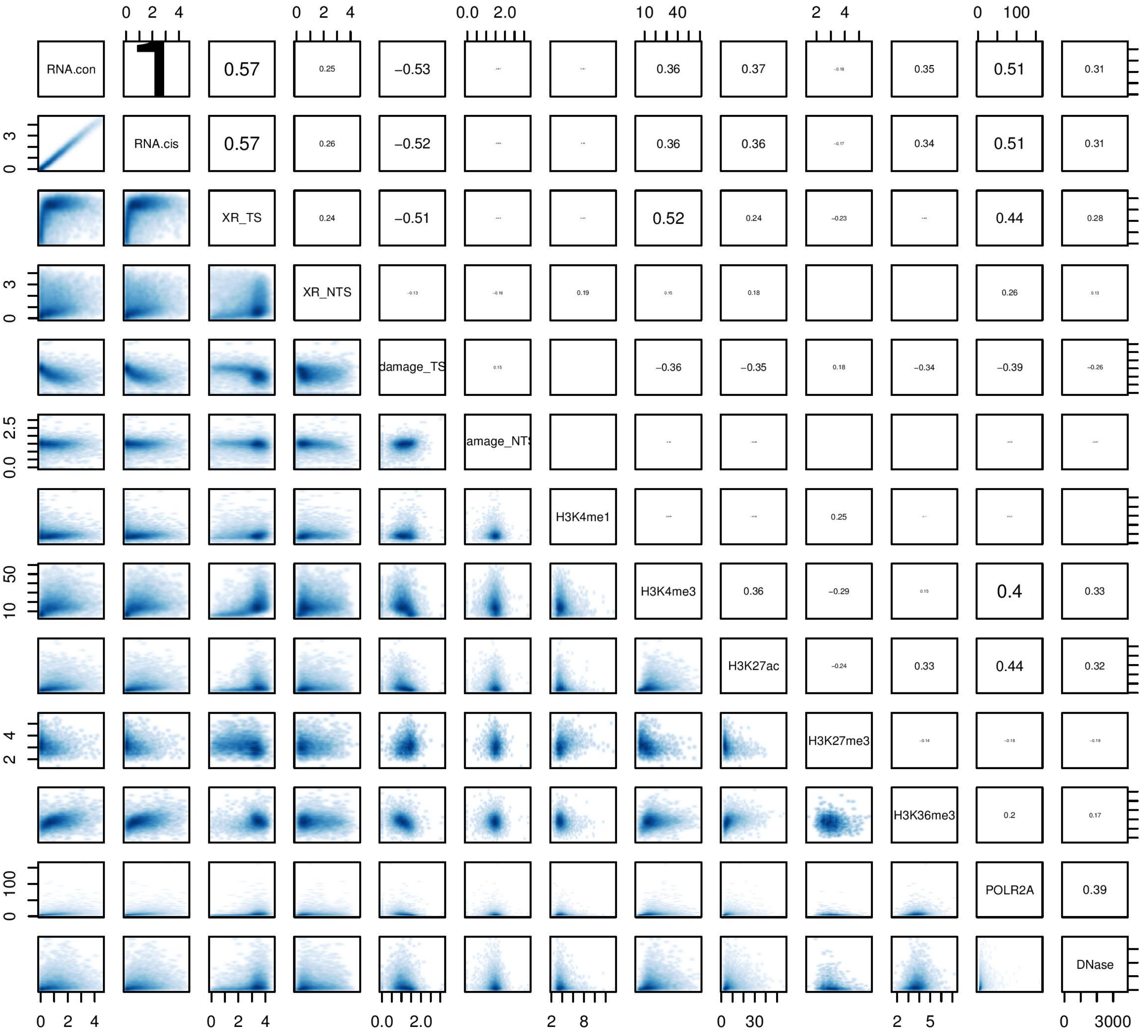

Supplementary Figure 17. Relationship between gene expression and epigenomic profiles in cisplatin samples. Pairwise scatterplots are shown, with the lower panel being the smooth scatterplots and the upper panel being the Spearman correlation coefficient for each pair of gene-specific measurements. Only genes that have insignificant differential expression after cisplatin treatment across all organs are plotted and included in the correlation analysis. Excision repair in the TS is positively correlated with gene expression, H3K4me3, and DNase I markers across all organs, while the damage in the TS is negatively correlated with the aforementioned epigenetic markers. **a** kidney. **b** liver. **c** lung. **d** spleen.

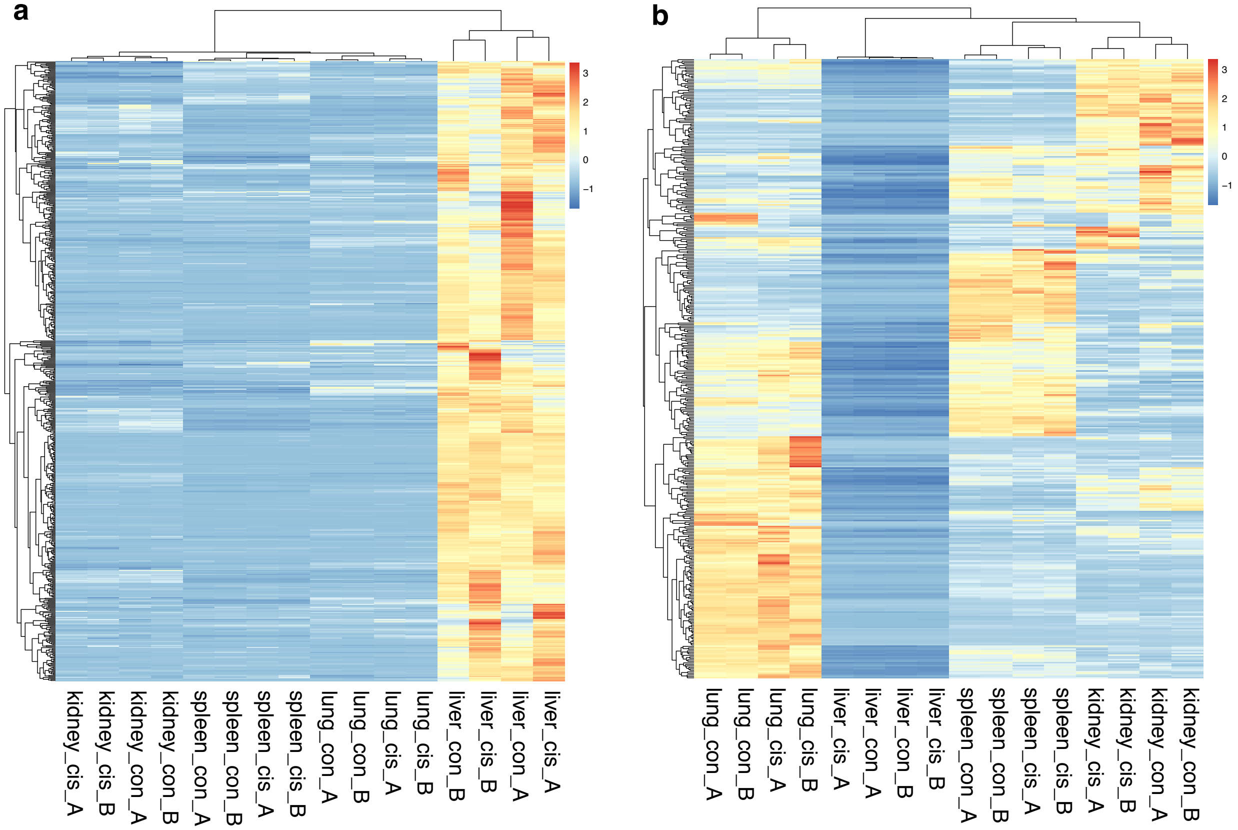

Supplementary Figure 18. Genes with significant high/low expression in liver compared to other organs. Pairwise differential expression analysis that compares liver with each other organ is carried out using DESeq2. The intersection of significant genes from the pairwise comparisons return the genes that are **a** highly and **b** lowly expressed in liver.

a

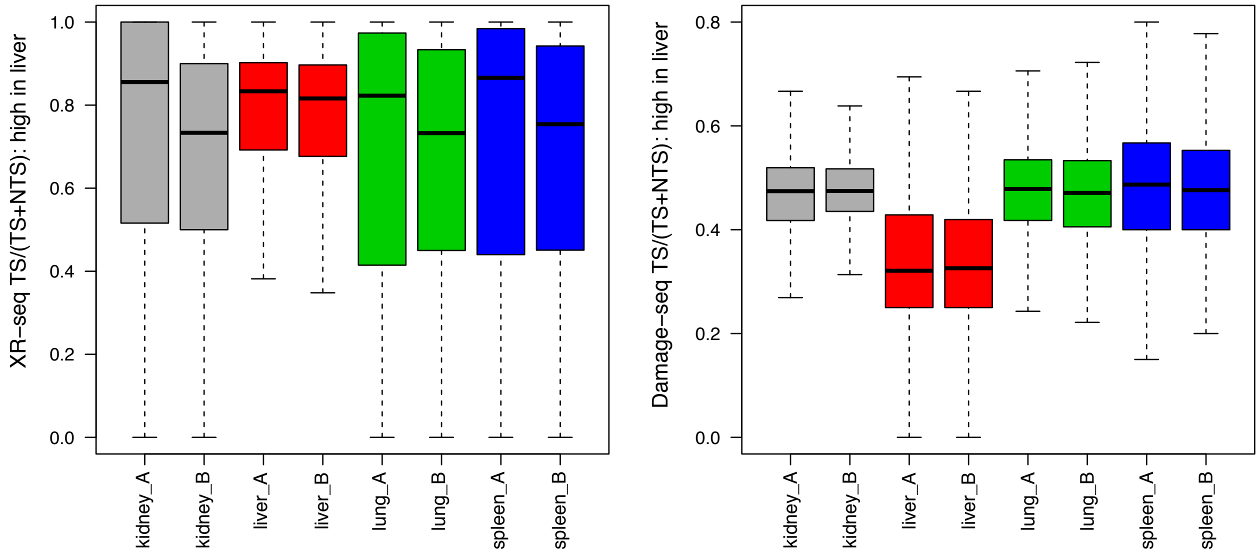

b

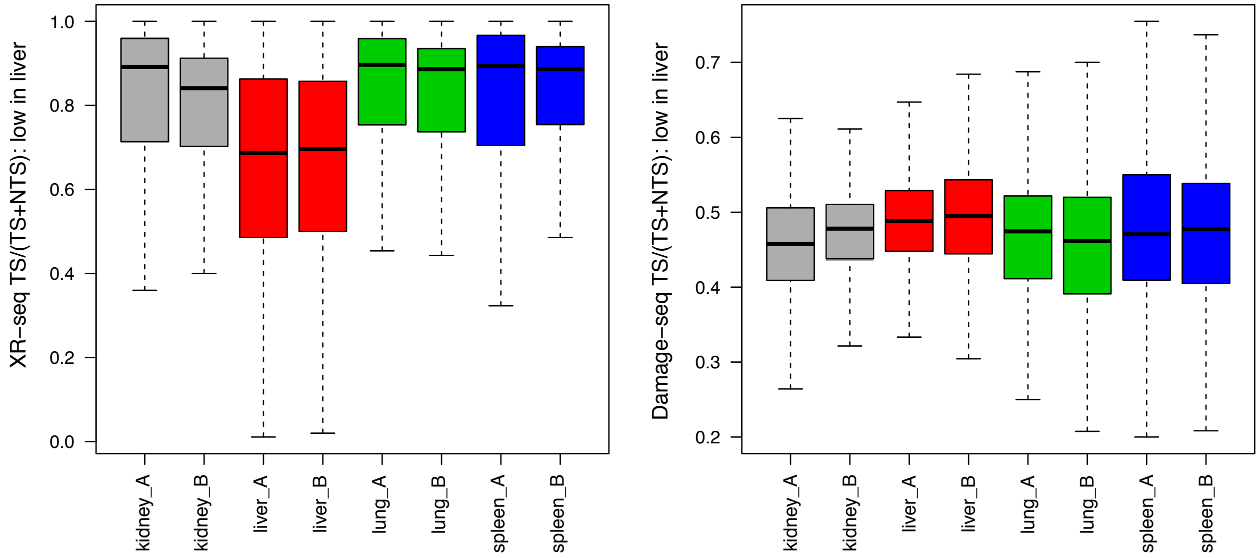

Supplementary Figure 19. TS/(TS+NTS) ratio for highly and weakly expressed genes in liver. The ratio is taken to remove biases and artifacts. **a** For genes that are highly expressed in liver, transcription-coupled repair is higher, resulting in less damage in the TS. **b** For genes that are weakly expressed in liver, transcription-coupled repair is lower, leaving damage level unchanged.

Supplementary Table 1. Publicly available epigenomic data from ENCODE. Epigenomic data for mouse organs were downloaded from the ENCODE portal (https://www.encodeproject.org) as bed files and viewed using the UCSC browser and the Integrative Genomics Viewer. Data accession number as well as age of mice are included for each marker across all organs.

Organ	H3K4me1	H3K4me3	H3K27ac	H3K27me3	H3K36me3	POLR2A	DNase I
Kidney	ENCFF764QKN (8 weeks)	ENCFF039JTO (8 weeks)	ENCFF660CQT (8 weeks)	ENCFF710FSC (8 weeks)	ENCFF996FJV (8 weeks)	ENCFF160ZYB (8 weeks)	ENCFF260THC (postnatal)
Liver	ENCFF189WSW (8 weeks)	ENCFF625DES (8 weeks)	ENCFF776JLM (8 weeks)	ENCFF429PBK (8 weeks)	ENCFF632JAR (8 weeks)	ENCFF125NGA (8 weeks)	ENCFF235KRN (postnatal)
Lung	ENCFF582IWI (8 weeks)	ENCFF543QNI (8 weeks)	ENCFF388BOS (postnatal)	ENCFF873ZSM (postnatal)	ENCFF176JQW (postnatal)	ENCFF460DBD (8 weeks)	ENCFF251HWU (postnatal)
Spleen	ENCFF817MPF (8 weeks)	ENCFF574OJR (8 weeks)	ENCFF092BMP (8 weeks)	ENCFF970VMF (8 weeks)	ENCFF362AJY (8 weeks)	ENCFF518LVB (8 weeks)	ENCFF553EPA (8 weeks)
