# Supplementary material for: Differential damage and repair of DNA-adducts induced by anti-cancer drug cisplatin across mouse organs
Source: Nat Commun. 2019 Jan 18;10:309. doi: 10.1038/s41467-019-08290-2 (PMC6338751; doi:10.1038/s41467-019-08290-2)
Supplement: Supplementary file 3 — Description of Additional Supplementary Files [file 41467_2019_8290_MOESM3_ESM.docx]

**Supplementary Data 1.** Sample information for Damage-seq, XR-seq, and RNA-seq. For Damage-seq and XR-seq, number of reads and proportion of reads out of the total are reported across all samples after each filtering step: total is for the total number of reads; mapq is for the reads with mapping quality greater than 20; chr is for the reads that are mapped to the autosomes and the sex chromosomes; GG is for the reads with GG dinucleotide sequence at the expected positions from 5’ end of the reads. For RNA-seq, assigned and unassigned reads are reported, respectively, along with the percentage of genes with non-zero read counts. Table is separately attached as an Excel file.

**Supplementary Data 2.** Differential expression analysis between cisplatin-treated group and control group. Results from DESeq2 are included for kidney, liver, lung, and spleen. Table is separately attached as an Excel file.

**Supplementary Data 3.** Canonical pathway analysis of differentially transcription data across organs. Ingenuity Pathway Analysis (IPA) on differentially expressed genes (adjusted p-value less than 0.05 and log_2_ fold change greater than 1 or less than -1). The most significant pathways across the mouse organs (kidney, liver, lung and spleen) are separately attached as an Excel file.
