# Supplementary material for: Differential damage and repair of DNA-adducts induced by anti-cancer drug cisplatin across mouse organs
Source: Nat Commun. 2019 Jan 18;10:309. doi: 10.1038/s41467-019-08290-2 (PMC6338751; doi:10.1038/s41467-019-08290-2)
Supplement: Supplementary file 7 — Reporting Summary [file 41467_2019_8290_MOESM7_ESM.pdf]

nature research

Corresponding author(s): *Double-blind peer review submissions: write DBPR and your manuscript number here instead of author names.*

Reporting Summary

Nature Research wishes to improve the reproducibility of the work that we publish. This form provides structure for consistency and transparency in reporting. For further information on Nature Research policies, see Authors & Referees and the Editorial Policy Checklist.

Statistical parameters

When statistical analyses are reported, confirm that the following items are present in the relevant location (e.g. figure legend, table legend, main text, or Methods section).

n/a	Confirmed	
☐	☒	The exact sample size (*n*) for each experimental group/condition, given as a discrete number and unit of measurement
☐	☒	An indication of whether measurements were taken from distinct samples or whether the same sample was measured repeatedly
☐	☒	The statistical test(s) used AND whether they are one- or two-sided<br>*Only common tests should be described solely by name; describe more complex techniques in the Methods section.*
☐	☒	A description of all covariates tested
☐	☒	A description of any assumptions or corrections, such as tests of normality and adjustment for multiple comparisons
☐	☒	A full description of the statistics including central tendency (e.g. means) or other basic estimates (e.g. regression coefficient) AND variation (e.g. standard deviation) or associated estimates of uncertainty (e.g. confidence intervals)
☐	☒	For null hypothesis testing, the test statistic (e.g. *F*, *t*, *r*) with confidence intervals, effect sizes, degrees of freedom and *P* value noted<br>*Give P values as exact values whenever suitable.*
☒	☐	For Bayesian analysis, information on the choice of priors and Markov chain Monte Carlo settings
☐	☒	For hierarchical and complex designs, identification of the appropriate level for tests and full reporting of outcomes
☐	☒	Estimates of effect sizes (e.g. Cohen's *d*, Pearson's *r*), indicating how they were calculated
☐	☒	Clearly defined error bars<br>*State explicitly what error bars represent (e.g. SD, SE, CI)*

Our web collection on statistics for biologists may be useful.

Software and code

Policy information about availability of computer code

Data collection	Microsoft Excel for Mac (v16.16.18111001), Linux system and R 3.4.0 GUI 1.70 El Capitan build.
Data analysis	Microsoft Excel, Adobe Illustrator CC 2018, Adobe Photoshop CC 2018, Cytoscape_v3.6.1, Ingenuity IPA-45868156, R 3.4.0 GUI 1.70 El Capitan build, REViGO, BWA, STAR, SAMtools, PicardTools, Cutadapt.

For manuscripts utilizing custom algorithms or software that are central to the research but not yet described in published literature, software must be made available to editors/reviewers upon request. We strongly encourage code deposition in a community repository (e.g. GitHub). See the Nature Research guidelines for submitting code & software for further information.

Data

Policy information about availability of data

All manuscripts must include a data availability statement. This statement should provide the following information, where applicable:

- Accession codes, unique identifiers, or web links for publicly available datasets
- A list of figures that have associated raw data
- A description of any restrictions on data availability

The data reported in this paper have been deposited in the Gene Expression Omnibus (GEO) database, accession number 'GSE117167'. All code used in this paper is available at https://github.com/yuchaojiang/damage_repair.

Field-specific reporting

Please select the best fit for your research. If you are not sure, read the appropriate sections before making your selection.

☒ Life sciences ☐ Behavioural & social sciences ☐ Ecological, evolutionary & environmental sciences

For a reference copy of the document with all sections, see nature.com/authors/policies/ReportingSummary-flat.pdf

Life sciences study design

All studies must disclose on these points even when the disclosure is negative.

Sample size	In animal studies, average sample sizes of 2-5 animals per group were deemed representative.
Data exclusions	No data points or animals were excluded in the analysis.
Replication	All experiments were repeated at least two biological replicates.
Randomization	No randommization was performed in this study.
Blinding	No blinding was performed in this study.

Reporting for specific materials, systems and methods

Materials & experimental systems

n/a	Involved in the study	
☒	☐	Unique biological materials
☐	☒	Antibodies
☒	☐	Eukaryotic cell lines
☒	☐	Palaeontology
☐	☒	Animals and other organisms
☒	☐	Human research participants

Methods

n/a	Involved in the study	
☒	☐	ChIP-seq
☒	☐	Flow cytometry
☒	☐	MRI-based neuroimaging

Antibodies

Antibodies used	All antibodies used in the study have been listed in Methods. Manufacturer, catalog number, species, dilution and application used are also included.
Validation	All antibodies used in the study are commercially available.<br>Anti-cisplatin antibody (ab 103261, Abcam), validation see: PMID: 29890208<br>Anti-DNA antibody (MAB 3034, Millipore Sigma), validation see: PMID: 25879486<br>Anti-TFIIH antibody p89 (G-10, Santa-Cruz), validation see: https://datasheets.scbt.com/sc-271500.pdf<br>Anti-TFIIH antibody p62 (H-10, Santa-Cruz), validation see: https://datasheets.scbt.com/sc-25329.pdf

Animals and other organisms

Policy information about studies involving animals; ARRIVE guidelines recommended for reporting animal research

Laboratory animals	Mice, C57BL/6J, Six or seven-month-old, were purchased from Jackson Laboratory (Bar Harbor, ME).
Wild animals	The study did not involve wild animals.
Field-collected samples	The study did not involve samples collected from field.
